# Essential role and therapeutic targeting of the glomerular endothelial glycocalyx in lupus nephritis

**DOI:** 10.1172/jci.insight.131252

**Published:** 2020-10-02

**Authors:** Hiroyuki Kadoya, Ning Yu, Ina Maria Schiessl, Anne Riquier-Brison, Georgina Gyarmati, Dorinne Desposito, Kengo Kidokoro, Matthew J. Butler, Chaim O. Jacob, János Peti-Peterdi

**Affiliations:** 1 Department of Physiology and Neuroscience and Department of Medicine, Zilkha Neurogenetic Institute, Keck School of Medicine, University of Southern California, Los Angeles, California, USA.; 2 Department of Nephrology/Hypertension, Kawasaki Medical School, Kurashiki, Japan.; 3 Division of Rheumatology, Department of Medicine, Keck School of Medicine, University of Southern California, Los Angeles, California, USA.; 4 Academic Renal Unit, School of Clinical Sciences, University of Bristol, Bristol, United Kingdom.

**Keywords:** Nephrology, Lupus, T cells, endothelial cells

## Abstract

Lupus nephritis (LN) is a major organ complication and cause of morbidity and mortality in patients with systemic lupus erythematosus (SLE). There is an unmet medical need for developing more efficient and specific, mechanism-based therapies, which depends on improved understanding of the underlying LN pathogenesis. Here we present direct visual evidence from high-power intravital imaging of the local kidney tissue microenvironment in mouse models showing that activated memory T cells originated in immune organs and the LN-specific robust accumulation of the glomerular endothelial glycocalyx played central roles in LN development. The glomerular homing of T cells was mediated via the direct binding of their CD44 to the hyaluronic acid (HA) component of the endothelial glycocalyx, and glycocalyx-degrading enzymes efficiently disrupted homing. Short-course treatment with either hyaluronidase or heparinase III provided long-term organ protection as evidenced by vastly improved albuminuria and survival rate. This glycocalyx/HA/memory T cell interaction is present in multiple SLE-affected organs and may be therapeutically targeted for SLE complications, including LN.

## Abstract 2

A combined immunology and renal pathophysiology study of the local kidney tissue microenvironment in lupus identifies a key role of glomerular endothelial glycocalyx in disease development.

## Graphical abstract

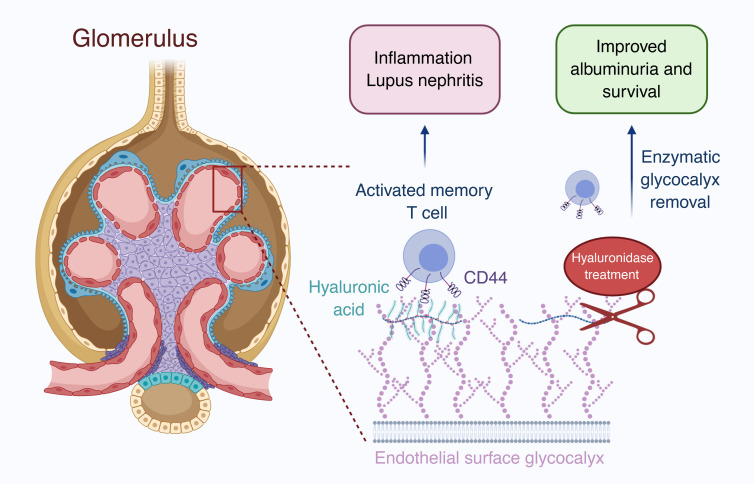

## Introduction

Systemic lupus erythematosus (SLE) is a chronic autoimmune disease affecting multiple organs in millions of patients worldwide. The development of kidney disease (lupus nephritis, LN) is a major complication affecting 60% of people with SLE and is the most important predictor of morbidity and mortality (1–3). With the gold standard being general immunosuppression, and with only a few emerging therapeutic options, the clinical management of LN remains unsatisfactory (1, 2). Highly efficient, targeted approaches need to be developed to improve the therapeutic efficiency in LN, which requires improved understanding of the rather complex LN pathogenic mechanism.

Over the years, extensive basic and clinical research efforts have focused on the role of immunoglobulin (autoantibody) and humoral mediators in defining pathogenic effector mechanisms of LN. Cellular effectors are more difficult to characterize and to study. Nonetheless, both the presence of effector T cells in lesions and their functional relevance in experimental models of glomerulonephritis imply a role for T cells in LN (1, 2). In addition to the immune system, there is growing recognition that local tissue-resident cells play key roles in pathogenic events in target tissues that are affected by SLE. In the kidney, podocytes, mesangial cells, and tubular epithelial cells have been implicated in LN development (4). The role of the vascular endothelium has been unknown, even though endothelial cells and the endothelial cell surface layer (also known as the glycocalyx) are the first point of contact with circulating immune system components.

To date, technical limitations have constituted a critical barrier in understanding the mechanistic details of LN and studying the kidney glomerulus, a critically important but generally inaccessible LN target tissue. Studying the individual glomerular cellular constituents in LN in vivo in their native environment has not previously been possible. Consequently, our knowledge on the dynamic structural and functional changes of the same glomerulus over time and the homing and interactions of infiltrating immune cells and resident cells in the intact living LN kidney is very limited. Clearly, there is need for new technology and research approaches to interrogate molecular and cellular interactions within the local kidney tissue microenvironment in LN.

Numerous modalities of intravital imaging with multiphoton microscopy (MPM), including serial MPM of the same glomerulus over time, have been developed recently, which allow insight into the biology of living renal cell types in the intact kidney and glomerulus in vivo in unprecedented detail (5, 6). In fact, MPM is the only current research technology that is capable of surveying the dynamics of the LN glomerular microenvironment with subcellular resolution.

Here we applied serial intravital MPM imaging in mouse models of LN to gain new direct visual clues on glomerular alterations and the key molecular and cellular players in LN pathogenesis, including fate tracking of immune cells. The present study aimed to test the hypothesis that an interplay between cellular components of the immune system (activated memory T cells) and local kidney tissue factors (the CD44 ligand hyaluronic acid [HA]) in the glomerular endothelial glycocalyx is critically important in the glomerular homing of T cells. We also hypothesized that disrupting immune cell binding to the glomerular endothelial surface layer by glycocalyx-removing enzymes improves LN.

## Results

### Activated memory T cell numbers in spleen correlate with LN disease progression in New Zealand mixed 2328 WT and genetically manipulated mice.

As shown in previous studies, the New Zealand mixed 2328 (NZM.2328) recombinant inbred mouse strain derived from extended intercross of the classic (NZB×NZW) F1 mice (7) is the closest model to the original F1 and probably to human LN (8, 9). Like humans, NZM.2328 female mice (but rarely males) slowly but progressively and fully develop lupus-like disease characterized by contribution of multiple genes (each with moderate effect), multiple types of autoantibodies, immune-regulatory changes in various immune cell subsets, and acute glomerulonephritis (GN) progressing to chronic GN (7), resembling that of patients with SLE.

**Figure 1:**
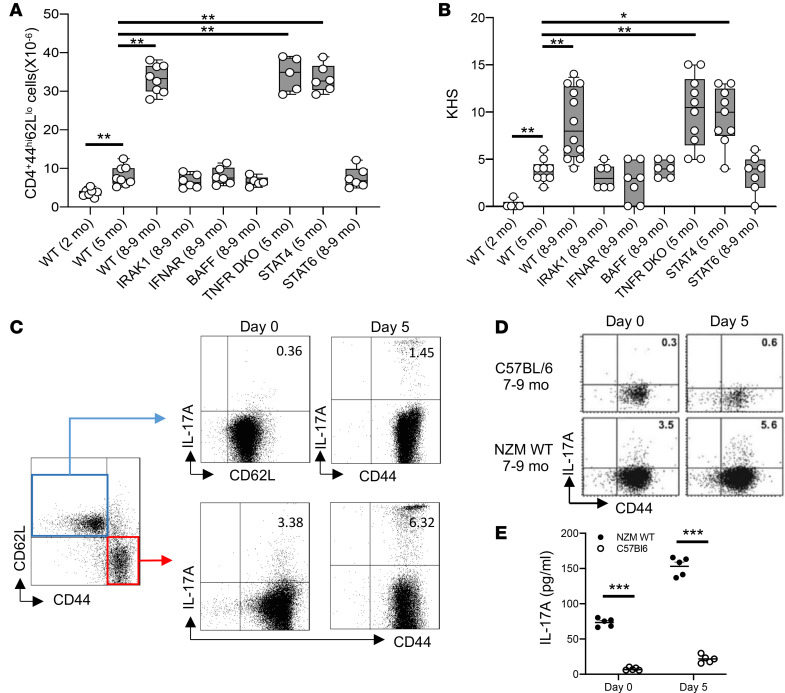
Accumulation of activated memory T cells in spleens correlate with LN disease progression in NZM.2328 mice. (**A**) Activated memory T cells were enumerated by flow cytometry from splenocytes purified from the different mouse lines at the ages indicated. Data are presented as box plots, where the boxes represent the 25th to 75th percentiles, the lines within the boxes represent the median, and the lines outside the boxes the 10th and 90th percentiles. Each circle represents an individual mouse. (**B**) Assessment of renal pathology in the different lines of mice. Data are presented as total kidney histology scores (KHSs) in the NZM.2328 mice with the indicated genotypes at the indicated ages. Each symbol represents an individual mouse. Results are plotted as in **A**. (**C**) Naive and activated memory T cells were stimulated with PMA and ionomycin for 5 hours and Brefeldin A (BFA) for 4 hours (day 0) or were stimulated with anti-CD3/CD28–coated beads for 5 days,and PMA + ionomycin were added to the culture for the last 5 hours as above and stained for intracellular IL-17A at day 0 and day 5. (**D**) Comparison between IL-17A production by activated memory T cells from age-matched NZM and C57BL/6 female mice stimulated as above. (**E**) IL-17A levels measured by commercial ELISA in supernatants from cultures of activated T cells isolated and stimulated as above. (**C** and **D**) Representative flow cytometry dot plots of 4 independent experiments are shown. (**D** and **E**) *n* = 5 per each group. (**A**–**E**) Only statistically significant differences are marked, based on using 1-way ANOVA followed by Tukey’s multiple-comparisons test (**A** and **B**) and unpaired Student’s *t* test (**E**). *P < 0.01, **P < 0.001, ***P < 0.0001.

Here we demonstrate that an activated memory T cell subset defined as CD3^+^CD4^+^CD62L^lo/neg^CD44^hi^ in spleens of NZM.2328 mice is a major biological marker of clinical LN disease progression. As shown in Figure 1A, there is a gradual (but statistically significant, *P* < 0.001) shift toward this T cell subset in NZM.2328 female mice from very low levels at 2 months old (pre-LN, no disease) to 5 months (subclinical disease) and with peak levels coinciding with full-blown clinical LN at 8–9 months of age. Transgenic manipulations of the NZM.2328 WT mice that cause accelerated LN, such as deletion of STAT4 (8), or TNF receptor p55/p75–double KO (TNFR-DKO) (10) mice show accelerated accumulation of activated memory T cells so that levels at 5 months of age are similar to the levels seen in NZM.2328 WT (NZM WT) mice at 8–9 months (Figure 1A). On the other hand, manipulation of NZM mice, such as deletion of IL-1 receptor-associated kinase 1 (IRAK1), type 1 IFN receptor A (IFNAR) (11), B cell activating factor (BAFF) (12), or STAT6 (8), that do not develop clinical LN show reduced levels of activated memory T cells at 8–9 months, to levels seen in WT at 5 months when there is no clinical disease. Furthermore, this subset of T cells exhibited a Th17.1 gene expression profile (Supplemental Figure 1; supplemental material available online with this article; https://doi.org/10.1172/jci.insight.131252DS1) and produced IL-17A (Figure 1, C–E).

Accumulated kidney dysfunction/scarring was summed up by obtaining a composite kidney histology score (KHS). Figure 1B shows the total KHS of the same mouse lines shown in Figure 1A. Mouse lines that develop accelerated clinical LN with high levels of proteinuria at much younger age, such as the TNFR-DKO and Stat4-KO lupus mice, show significantly greater composite KHS (*P* < 0.001) at 5 months than NZM WT at 5 months or lupus mouse lines that show diminished LN. Evaluated together, Figure 1, A and B, demonstrate that the abundance of activated memory T cell subset correlates with the accrued structural kidney dysfunction evaluated by the KHS. Spearman’s correlation analysis between the KHS and the number of splenic activated memory T cells showed a highly significant correlation, with *r* = 0.783 and *P* = 0.009.

Importantly, the correlation between this subset of T lymphocytes and lethal LN is much stronger and more consistent than levels of autoantibodies (including the LN-associated anti-dsDNA autoantibodies) (8) or histological evidence of IgG and complement deposition in kidney (12, 13).

### Enumeration and characterization of immune cells infiltrating the kidney of NZM WT mice.

**Figure 2:**
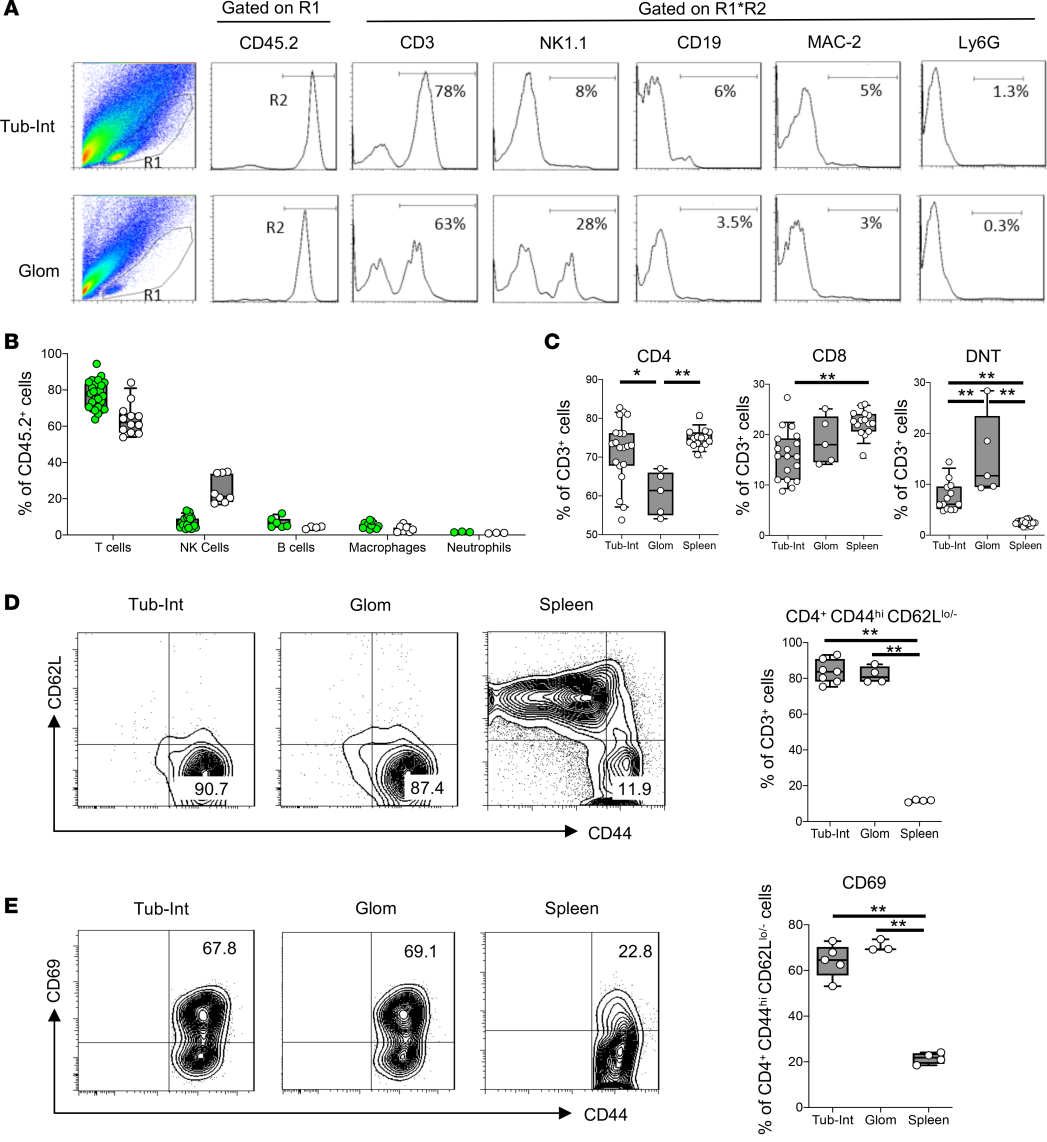
Enumeration and characterization of leukocytes infiltrating the kidneys of 6- to 7-month-old female NZM.2328 WT mice. (**A**) Representative flow cytometry plots of infiltrating leukocyte subsets in the glomerular (glom) and tubulointerstitial (Tub-int) kidney compartments. (**B**) Flow cytometry–based quantification of the indicated leukocyte subsets infiltrating the glom (white symbols) and the Tub-int (green symbols) compartments. Each symbol represents an individual mouse. (**C**) Comparison of CD4^+^, CD8^+^, and CD4^–^CD8^–^ T cells (double-negative T cells, DNT) presented as percentage of CD3^+^ cells in kidney compartments and spleens. Data are presented as box plots as in Figure 1. (**D**) Representative flow cytometry contour plots and quantification of activated memory T cells (presented as % of CD3^+^) in kidney compartments and spleens. (**E**) Representative contour plots and quantification of % CD69^+^ cells on activated memory T cells in kidney compartments and spleens. (**B**–**E**) Each symbol represents an individual mouse. Only statistically significant differences are marked, based on using 1-way ANOVA followed by Tukey’s multiple-comparisons test (**C**–**E**). *P < 0.01, **P < 0.001.

To gain insight into the functional importance of this T cell subset in LN pathogenesis, we first characterized the composition of infiltrating immune cells in kidneys of LN mice. Infiltrating immune cells from glomeruli and independently from the tubulointerstitial compartments were isolated from mouse kidneys using the methodology described and validated by Takemoto et al. (14). We have systematically quantified and characterized these cells from NZM.2328 WT lupus mice using flow cytometry during different stages of disease development. Figure 2, A–E, show the cellular profile of infiltrating immune cells. Approximately 80% and 65% of the infiltrating cells into tubulointerstitial compartments and glomeruli, respectively, were T cells already by the age of 6–7 months (Figure 2, A and B). NK cells represented approximately 25% of glomerular infiltrate and up to 10% of tubulointerstitial infiltrate. B cells and macrophages comprised approximately 5% each of the infiltrate (Figure 2, A and B). The infiltrating T cells, whether CD4^+^, CD8^+^, or double negative (CD4^–^CD8^–^) (Figure 2C), had an activated memory phenotype (Figure 2D) and expressed the activation marker CD69 (Figure 2E). While this subset represented no more than 20% of splenic T cells, in the kidney over 80% showed this phenotype (Figure 2, D and E).

### Rapid progressive lupus mouse model for in vivo MPM imaging.

**Figure 3:**
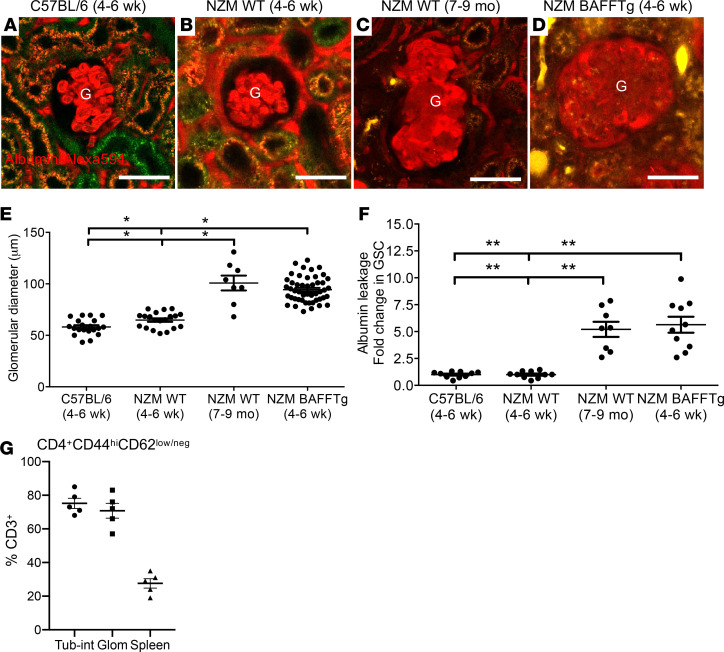
Evaluation of the phenotype of NZM.BAFFTg mice by MPM imaging and cytometry. Intravascular space (plasma) was labeled with intravenous-injected (iv-injected) albumin–Alexa Fluor 594 (red). (**A**–**D**) Representative images of glomeruli in C57BL/6, NZM WT (4–6 weeks and 7–9 months), and NZM.BAFFTg (4–6 weeks) mice. Scale bar: 50 μm. Summary of (**E**) glomerular diameter evaluating hypertrophy (*n* = 8–48 glomeruli from *n* = 4 mice each group) and (**F**) glomerular sieving coefficient (GSC) evaluating albumin leakage into Bowman’s space (*n* = 8–10 glomeruli from *n* = 4 mice each group). (**G**) Quantification of activated memory T cells (presented as % of CD3^+^) in the tubulointerstitial (Tub-int) and glomerular (Glom) kidney compartments and spleen. Data are expressed as means ± SEM. *P < 0.05, **P < 0.01 using 1-way ANOVA followed by Tukey’s multiple-comparisons test (**E** and **F**).

We have produced BAFF-transgenic mice on the NZM.2328 background (NZM.BAFFTg). These mice overexpress murine BAFF driven by a liver-specific alpha1-antitrypsin promoter (15). Approximately 50% of these transgenic mice developed spontaneous LN by 3–4 weeks of age, as evidenced by the sudden appearance of proteinuria and glomerular albumin leakage (Figure 3), and succumbed to renal failure within approximately 2–3 weeks thereafter. Because these young mice feature a rapid disease process and exhibit many superficial glomeruli that can be directly visualized by MPM imaging, they present a unique opportunity to track changes in the glomerular microenvironment during disease progression and to quantitatively visualize the effect of therapeutic intervention in the intact LN mouse kidney in vivo. MPM imaging confirmed significant glomerular hypertrophy and albumin leakage in these NZM.BAFFTg mice (Figure 3, A–F). Figure 3G shows the proportion of activated memory T cells in kidney compartments and spleens of NZM.BAFFTg mice. They are similar to the proportion of such cells in the spleen and kidney compartments of nephritic NZM WT mice (Figure 2D), thus linking the acute model to the original chronic model.

### MPM imaging of the glomerular homing of iv-injected, FACS-sorted, splenic activated memory T cells.

**Figure 4:**
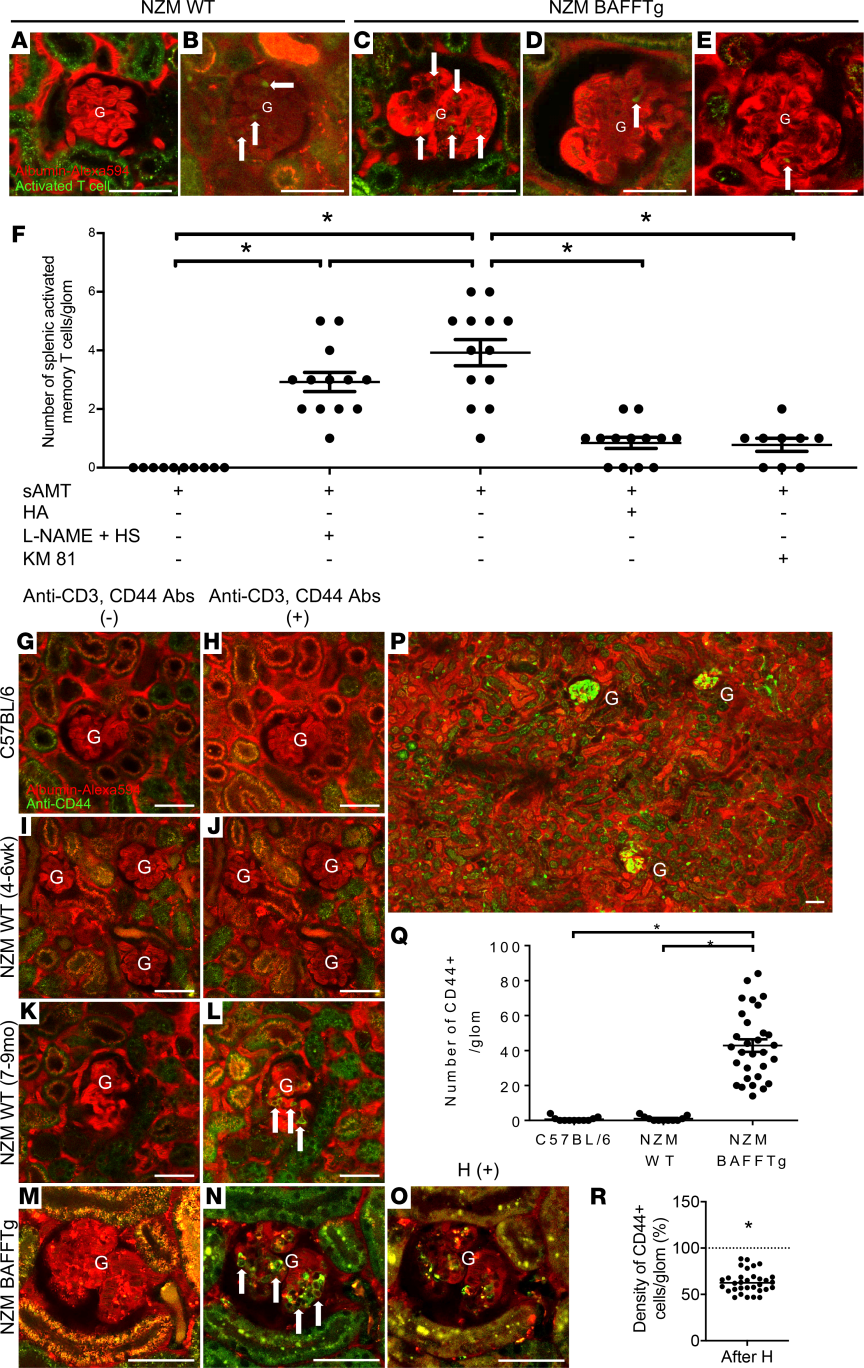
In vivo MPM imaging of the glomerular homing of exogenous and endogenous activated memory T cells. Intravascular space (plasma) was labeled with iv injected albumin–Alexa Fluor 594 (red). Female 4- to 6-week-old NZM WT (**A** and **B**) and NZM.BAFFTg (**C**–**E**) mice were treated with l-NAME and high-salt diet (HS) (**B**) or vehicle (**A** and **C**) followed by iv injection of FACS-sorted splenic activated memory T cells (sAMTs) (**A**–**C**). All transferred activated memory T cells were obtained from 7- to 9-month-old NZM.2328 female mouse donors. (**D**) sAMTs were preincubated with HA before iv injection. (**E**) sAMTs were preincubated with anti-CD44 antibody (KM81) before iv injection. (**F**) Summary of the number of sAMTs homed in glomeruli of the different groups of mice (*n* = 9–13 glomeruli from *n* = 4 mice for **A**–**D** and *n* = 3 mice for **E**). (**G**–**Q**) Fluorescence labeling and MPM imaging of endogenous CD3^+^CD44^+^ T cells, in C57BL/6 (*n* = 3), NZM WT (*n* = 3), and NZM.BAFFTg (*n* = 10) mouse glomeruli (4–6 weeks old) and NZM WT (7–9 months, *n* = 1). (**G**, **I**, **K**, and **M**) Representative images at baseline before and (**H**, **J**, **L**, and **N**) after endogenous T cells labeled in same glomeruli. (**O**) Image of the same glomerulus as in **N** 1 hour after H injection. (**P**) Overview image of a large kidney surface area in a 4-week-old female NZM.BAFFTg mouse. Note the high density of endogenous T cells (green) in 3 glomeruli (G) compared with adjacent tubulointerstitial areas. (**Q**) Summary of the number of CD3^+^CD44^+^ T cells homed in glomeruli of different mouse groups at 4–6 weeks. (**R**) Normalized density of CD44^+^ cells homed to glomeruli after acute H injection, compared with baseline (dotted line at 100%). Scale bars: 50 μm. Values are expressed as means ± SEM, *P < 0.0001 based on using 1-way ANOVA followed by Tukey’s multiple-comparisons test (**F**–**Q**) and unpaired Student’s *t* test (**R**). Positive cells are depicted by white arrows. HA, hyaluronic acid; H, hyaluronidase.

Using intravital MPM, we show that freshly sorted splenic activated memory T cells derived from spleens of 7- to 9-month-old donor NZM WT female mice, injected iv, are present in the circulating blood in the intact kidney and home into affected glomeruli of NZM.BAFFTg kidneys in vivo (Figure 4, C and F) but not into glomeruli of healthy 4- to 6-week-old NZM WT mice in the absence of any kidney pathology (pre-LN stage) (Figure 4, A and F). Memory T cell homing could be induced in glomeruli of NZM WT mice with the nitric oxide synthase inhibitor l-NG-nitroarginine methyl ester (l-NAME) and high-salt diet treatment (Figure 4, B and F), which has been used widely to induce endothelial dysfunction (16). Indeed, the combined l-NAME and high-salt treatment of NZM WT (4–6 weeks old) mice for 2 weeks induced glomerular homing of memory T cells in these mice, similarly to NZM.BAFFTg mice (Figure 4, B and F; and Supplemental Figure 2). To test the specificity of binding via HA, we preincubated freshly sorted splenic activated memory T cells with a saturating dose of HA before iv injection into NZM.BAFFTg mice. As shown in Figure 4, D and F, a short incubation with HA significantly reduced the number of activated memory T cells homed into affected glomeruli, supporting the notion that CD44 on activated memory T cells binds HA (17) in the glycocalyx of glomerular endothelial cells. Moreover, we evaluated CD44-HA interaction by preincubating freshly sorted splenic activated memory T cells with CD44-specific blocking monoclonal antibodies (mAbs) before iv injection. Anti-CD44 mAbs (KM81) blocked the glomerular homing of these cells (Figure 4, E and F) in a dose-dependent manner (Supplemental Figure 3).

### Fluorescence labeling and MPM imaging of endogenous CD3^+^CD44^+^ T cells and the effects of hyaluronidase treatment.

In addition to imaging exogenous (FACS sorted from donor LN mice), iv-injected memory T cells, we visualized and followed the fate of endogenous activated memory T cells in vivo by iv injection of fluorochrome-conjugated anti-CD3 and anti-CD44 mAbs (Figure 4, G–R). On average, we observed 43 ± 4 CD3^+^CD44^+^ T cells per glomerulus (Supplemental Video 1) sticking to endothelial cells within glomerular capillaries of NZM.BAFFTg mice (Figure 4, N, P, and Q; and Supplemental Video 2) but none in C57BL/6 or NZM WT (4–6 weeks) mice (Figure 4, G, I, and Q). Endogenous T cells showed preferential glomerular rather than tubulointerstitial homing in NZM.BAFFTg mice (Figure 4P), although the latter was also clearly detectable (Supplemental Figure 4 and Supplemental Video 1). We have confirmed that almost all CD44^+^ cells in glomeruli were also positively labeled for CD3 (Supplemental Figure 4), supporting the notion that the in vivo–labeled cells were activated memory T cells. Although clinically nephritic 8- to 9-month-old NZM WT mice had few to no superficial glomeruli for MPM imaging, in the few cases where such glomeruli were identified, we observed glomerular homing of endogenous CD3^+^CD44^+^ T cells in an extent similar to what was found in NZM.BAFFTg mice (Figure 4, K and L).

Because the glomerular endothelial surface layer is the first point of contact with circulating immune cells, we tested the effects of the glycocalyx-degrading enzyme hyaluronidase (H). A key finding was the major effect of a single iv injection of the enzyme H, which significantly reduced (by ~40%) the number of homed CD3^+^CD44^+^ cells in the same glomeruli, within 1 hour of injection (Figure 4, O and R; and Supplemental Video 3).

### Quantitative MPM imaging of the glomerular endothelial glycocalyx.

**Figure 5:**
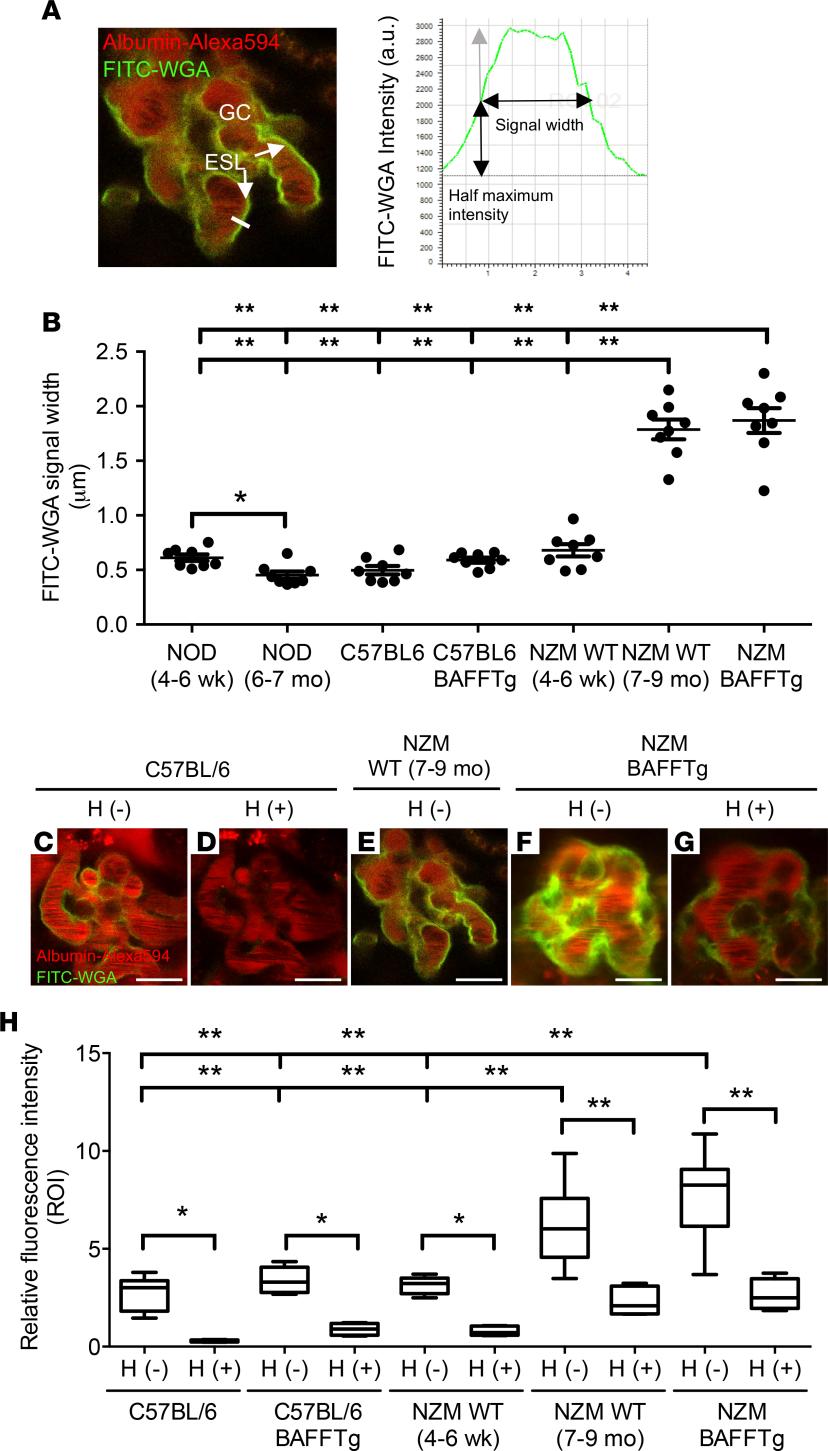
Quantitative MPM imaging of the glomerular endothelial glycocalyx in different mouse models using iv-injected FITC-WGA lectin (shown in green). Intravascular space (plasma) was labeled with iv-injected albumin–Alexa Fluor 594 (red). (**A**) Representative image and calculation of endothelial surface FITC-WGA signal width on linear intensity profiles as an index of glycocalyx thickness. FITC-WGA fluorescence decreases as it meets the plasma (luminal end) and the endothelial cell (abluminal end). FITC-WGA signal width was measured as the distance between half maximal intensities as shown. Glycocalyx measurements relied on the selection of capillary segments where clear plasma and FITC-WGA peak signals could be seen. Only clear longitudinal or cross-sectioned (round) capillary profiles and the level at one-half the capillary depth (deduced from the *Z*-stack) were analyzed to ensure the glycocalyx was measured perpendicular to the endothelial membrane. (**B**) Summary of FITC-WGA signal width in various mouse groups as shown. (**C**–**G**) Representative images of FITC-WGA lectin labeling and intensity before and 1 hour after H treatment. (**H**) Summary and statistical analysis of FITC-WGA fluorescence intensity (ratio of glomerular labeling/proximal tubule autofluorescence). (**B**–**H**) Mice were 4–6 weeks old unless stated otherwise. Scale bar: 10 μm. *n* = 8 glomeruli from *n* = 4 mice each group. Values are expressed as means ± SEM, *P < 0.005, **P < 0.001, based on 1-way ANOVA followed by Tukey’s multiple-comparisons test (**B**–**H**). GC, glomerular capillary; ESL, endothelial surface layer; NOD, nonobese diabetic; H, hyaluronidase.

The l-NAME experiments (Figure 4 and Supplemental Figure 2) suggested that glomerular endothelial cell alterations may have a causal effect on the homing of activated memory T cells. Therefore, we analyzed the glomerular endothelial glycocalyx and its specific molecular component, the CD44 ligand HA (17). Injection of FITC-labeled wheat germ agglutinin (FITC-WGA) lectin iv was used to visualize and measure glomerular endothelial surface glycocalyx thickness as evaluated by the FITC-WGA signal width and intensity (18, 19) before and after H treatment. Strong accumulation of endothelial glycocalyx in an even, linear pattern along the endothelial surface was observed in mice with clinical LN, namely NZM.BAFFTg mice and NZM WT (7–9 months old), but not in healthy 4- to 6-week-old pre-LN NZM WT mice or nonautoimmune C57BL/6 WT or C57BL/6.BAFFTg mice (Figure 5, A–C, E, F, and H; and Supplemental Figure 5A and Supplemental Video 4). While the thickness of the glomerular endothelial surface glycocalyx in healthy mice was 0.5~0.6 μm, in the nephritic LN NZM.BAFFTg and 7- to 9-month-old NZM WT mice, the thickness increased to 1.7–1.9 μm. The accumulation of endothelial glycocalyx in LN appeared to be specific for the glomerular capillaries compared with normal levels found in peritubular capillaries in the renal cortex (Supplemental Figure 5A) and was not observed in the autoimmune nonobese diabetic (NOD) mouse model. In fact, glycocalyx thickness was decreased in 6- to 7-month-old NOD mice compared with young 4- to 6-week-old NOD mice (*P* < 0.005) (Figure 5B and Supplemental Figure 5E). In older NOD mice, the decreased glomerular endothelial glycocalyx was associated with a substantial increase in glomerular albumin permeability to levels that reflected macroalbuminuria states (20, 21) (Supplemental Figure 5F). H injection significantly diminished FITC-WGA lectin fluorescence intensity in all mice (Figure 5, D, G, and H; Supplemental Figure 5A; and Supplemental Video 5). Even with a thickened glycocalyx, glomerular capillary blood flow was preserved in mice with LN, as indicated by the free passage of fast streaming red blood cells in the capillary lumen (Figure 5 and Supplemental Video 4).

### Quantitative measurement of the specific HA content of the glomerular endothelial glycocalyx.

**Figure 6:**
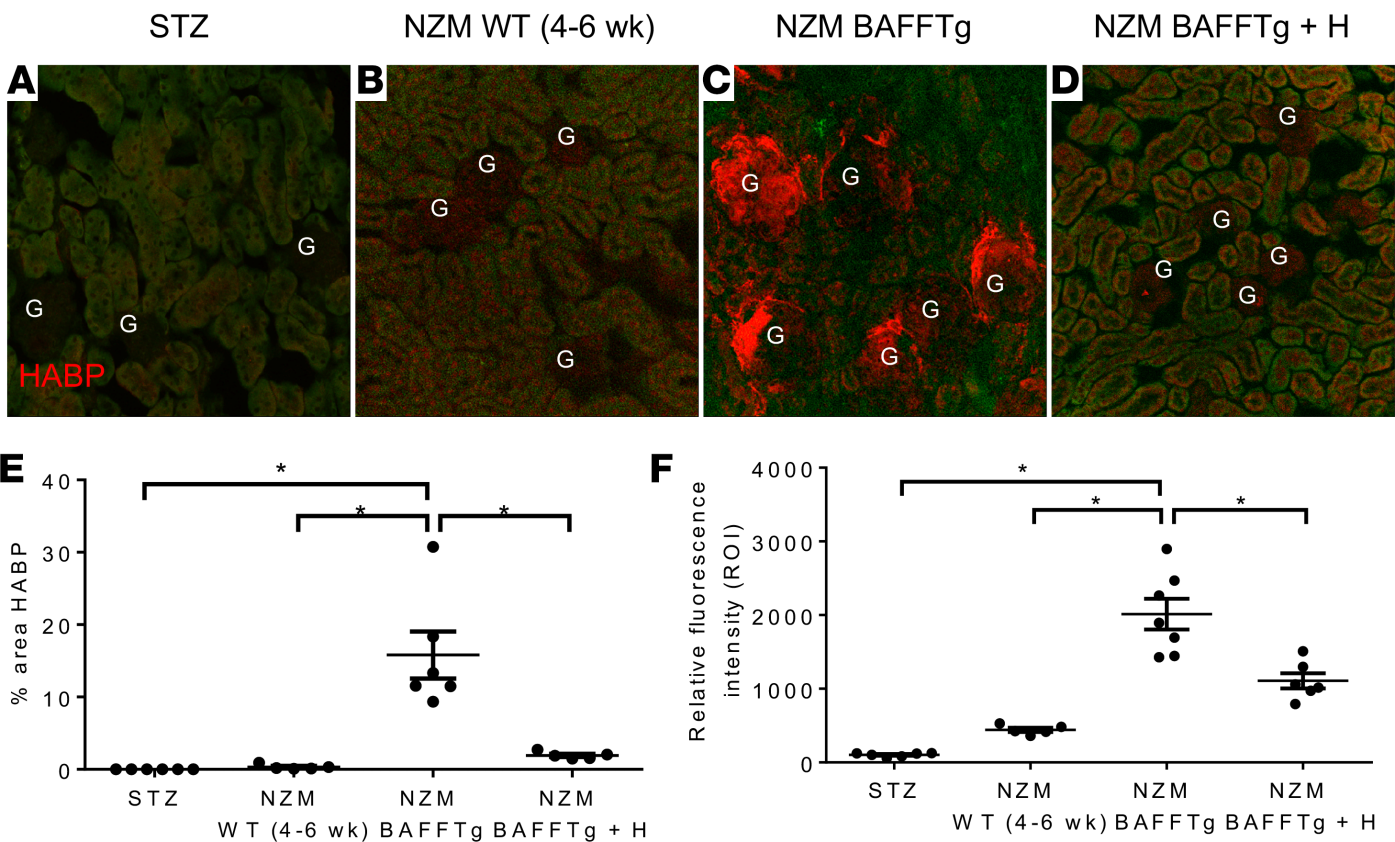
Quantitative evaluation of the specific HA content of the glomerular endothelial glycocalyx in various mouse models. (**A**–**D**) Representative images of Alexa Fluor 594–conjugated HABP (red) labeling of fixed kidney sections. (**E** and **F**) Summary and statistical analysis of percentage area of HABP labeling (**E**) and HABP fluorescence intensity (**F**) in *n* = 6 glomeruli from *n* = 3 mice in each group. Scale bar: 50 μm. Values are expressed as means ± SEM. *P < 0.0001, based on 1-way ANOVA followed by Tukey’s multiple-comparisons test (**E** and **F**). G, glomeruli; H, hyaluronidase; STZ, streptozotocin-induced diabetic mice.

The specific HA content of glomerular endothelial glycocalyx was labeled with Alexa Fluor 594–conjugated HA binding peptide (22) (HABP) on frozen tissue sections and then quantified. While HA labeling was below the limits of detection in streptozotocin-induced (STZ) diabetic or NZM WT (4–6 weeks) mice, robust accumulation of HA in the glomerular endothelial surface layer was evident in clinically nephritic NZM.BAFFTg mice (Figure 6, A–C, E, and F). Efficient removal of glomerular HA content by H treatment was confirmed by diminished HABP labeling in glomeruli (Figure 6, D–F). In addition to glomeruli, strong HABP labeling was observed around large blood vessels (Supplemental Figure 5G).

### The effects of H treatment on kidney and mouse LN phenotype.

**Figure 7:**
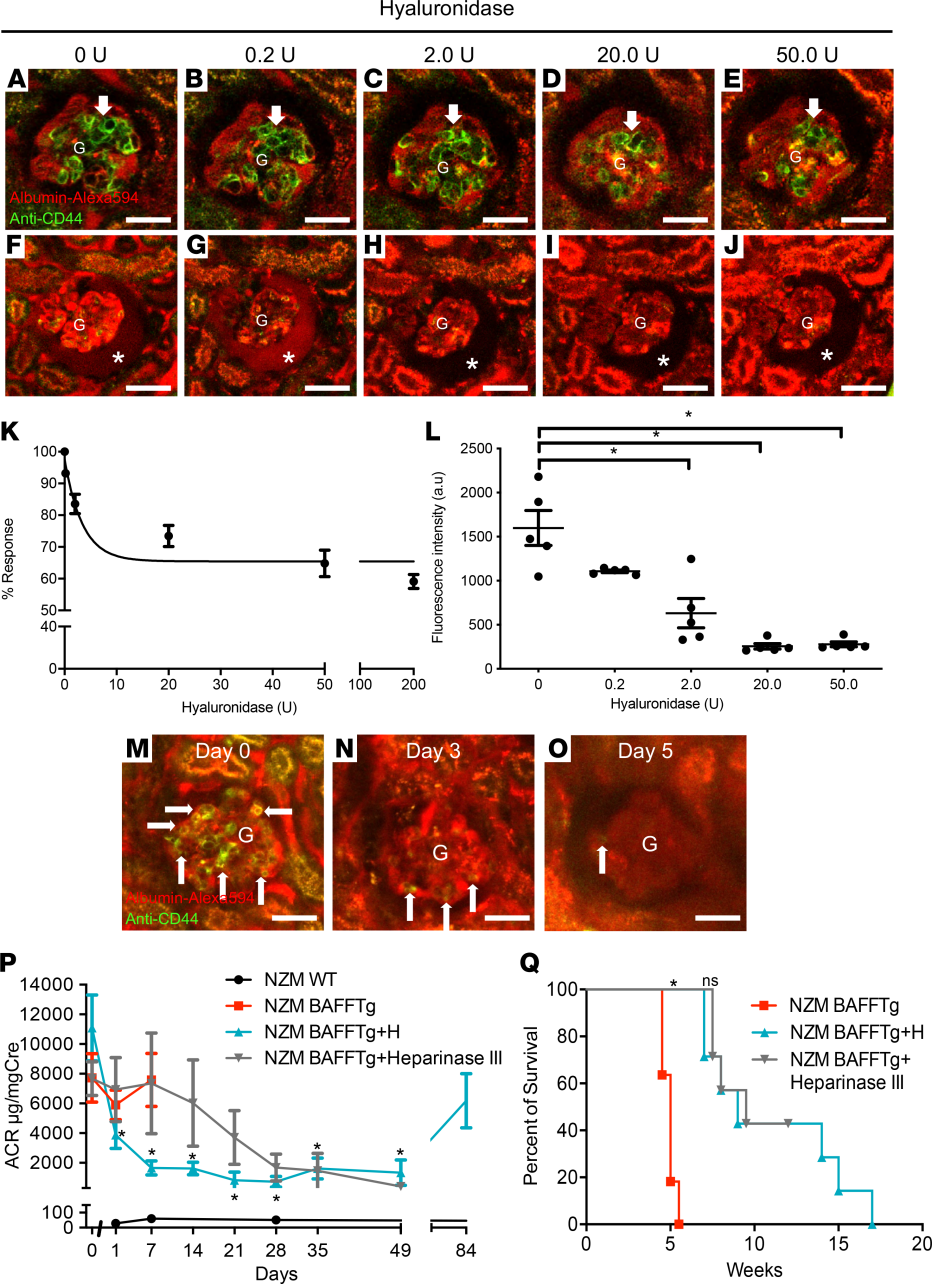
Fluorescence labeling and in vivo MPM imaging of endogenous CD3^+^CD44^+^ T cells and the effects of H treatment (0–50 U iv) in the same glomeruli of NZM.BAFFTg mice. Intravascular space (plasma) was labeled with iv-injected albumin–Alexa Fluor 594 (red). (**A**–**E**) Representative images of endogenous T cells (arrows) labeled with iv-injected Alexa Fluor 488–conjugated anti-CD44 (green) and Alexa Fluor 594–conjugated anti-CD3 mAb (red) before and after H injection. All scale bars are 50 μm. We have confirmed that practically all CD44^+^ cells in glomeruli were also CD3^+^ (Supplemental Figure 4). (**F**–**J**) Representative images of albumin leakage into Bowman’s space (asterisk). (**K**) Dose-response curve of H effect on reducing the number of homed T cells in glomeruli and (**L**) Bowman’s space albumin fluorescence intensity indicating glomerular albumin leakage (*n* = 5 glomeruli from *n* = 3 mice each). (**M**–**Q**) Effects of H treatment (3 times 200 U each iv every other day) in NZM.BAFFTg mice. Representative images (**M**–**O**) of the same glomerulus before H treatment (day 0) and after the second (day 3) and third H injections (day 5). (**P**) Control time course and the effects of H or heparinase III treatment on albuminuria (urinary albumin/creatinine ratio, ACR) in NZM WT and NZM BAFFTg mice followed for 3 months. Values are expressed as means ± SEM, *n* = 6 mice each. *P < 0.001. (**Q**) Kaplan-Meier animal survival curve. *P < 0.001 BAFFTg vs. treatment; ns, not significant H vs. heparinase III treatment. One-way ANOVA followed by Tukey’s multiple-comparisons test was used (**L**–**Q**).

In NZM.BAFFTg mice with active LN, the injection of H (0–50 U iv) reduced the number of intrinsic CD3^+^CD44^+^ T cells (Figure 7, A–E) and in parallel reduced the level of albumin leakage through the glomerular filtration barrier in a dose-dependent manner (Figure 7, F–J, and L). The dose-response relationship with an IC_50_ of about 20 U (Figure 7K) and the identical effects of 2 sources of H enzyme formulations (MilliporeSigma and Worthington, data not shown) suggest that the effects of H are specific to glomerular endothelial glycocalyx removal, blockade of T cell homing, and glomerular albumin leakage. Treatment with heparinase III, another glycocalyx-degrading enzyme, was also efficient in glycocalyx removal, as indicated by diminished FITC-WGA lectin labeling even 10 days after treatment (Supplemental Figure 5, B–D).

Next, we evaluated the long-term effects of short-course H treatment (a total of 3 iv injections given every other day, days 1, 3, 5) in NZM.BAFFTg mice (Figure 7). We successfully followed the same glomeruli before and after H treatment using serial intravital MPM imaging and confirmed that CD3^+^CD44^+^ T cells in glomeruli were almost completely eliminated by day 5 (Figure 7, M–O; and Supplemental Videos 6–8). Figure 7P shows longitudinal evaluation of albuminuria in these mice followed for up to 3 months and Figure 7Q shows their survival curve. While untreated mice died within a few days, H-treated mice showed cumulative reduction of albuminuria at least until day 50, and then albuminuria started to increase so that approximately 2.5 months after short-course H treatment, the levels of proteinuria were similar to those before treatment (Figure 7, P and Q). Identical short-course treatment with heparinase III had a similar protective effect on albuminuria and survival (Figure 7, P and Q). To further confirm the HA-degrading efficiency of the above repeated, short-course, systemic H treatment, plasma HA levels were measured using ELISA in pre-LN and LN mice before and after H treatment. Plasma HA levels were significantly increased in both NZM.BAFFTg and 7- to 9-month-old NZM WT mice compared with young NZM WT mice, while at the end of short-course H treatment, plasma HA levels returned to baseline (Supplemental Figure 5H). H treatment had no major direct effect on activated memory T cells isolated from 7- to 9-month-old female NZM.2328 WT mice, based on the finding of unaltered levels of IL-17A and retinoid-related orphan receptor–γ (RORgc) RNA measured in these cells after in vitro H treatment (50 U). The relative expression of IL-17A was 384 ± 17 before and 385 ± 14 after H treatment, while that of RORgc was 557 ± 21 and 563 ± 26 after H treatment (*n* = 6 each, data not shown).

In addition to glomeruli, intravital MPM confirmed the accumulation of activated memory T cells in other SLE target organs (e.g., skin, skeletal muscle, peritoneal membrane, spleen). As in the kidney, single iv injections of H efficiently blocked the homing of CD3^+^CD44^+^ T cells in these organs (Supplemental Figure 6A). Quantitative imaging of the endothelial surface layer in the spleen using FITC-WGA lectin labeling and MPM imaging showed slightly increased glycocalyx thickness in NZM.BAFFTg mice compared with young NZM WT mice. However, this increase did not reach statistical significance (Supplemental Figure 6B).

## Discussion

The present study combined immunology and renal pathophysiology research approaches to investigate the pathogenesis of LN in vivo in mouse models. The successful application of intravital MPM imaging of the local glomerular tissue microenvironment was a potentially novel and essential tool to obtain new visual clues on the key cellular and molecular players and to establish our main findings: (a) the glomerular homing of activated memory T cells and (b) the LN-specific, robust accumulation of the glomerular endothelial glycocalyx played central roles in LN development and that (c) treatment with glycocalyx-degrading enzymes vastly improved clinical LN and survival.

We present direct evidence for a mechanistically crucial interplay between cellular components of the immune system and local kidney tissue factors in the pathogenesis of LN. Thus, we demonstrate that activated memory T cells may be the best indicator for LN development in spontaneous lupus mice (Figure 1). This subset of T cells that were originally generated in immune organs and propagated during disease development (23, 24) subsequently circulated in blood vessels and finally homed to clinically important organs affected in SLE, particularly the kidneys. We show that in NZM WT lupus mice this subset of T cells represent over 80% of T cells in the kidney even before the appearance of clinical LN, while they represent no more than 20% of T cells in the spleen of the same mice (Figure 2D). Moreover, activated memory T cells from NZM WT lupus mice produce the proinflammatory cytokine IL-17A (Figure 1, B–D; and Supplemental Figure 1). Importantly, such IL-17A–producing T cells have been demonstrated in kidney biopsies of people with SLE (25). Because the subset of IL-17–producing cells coexpress IFN-γ (Supplemental Figure 1), they cannot be considered “pure” Th17 cells, but rather are called Th17.1 cells (26). Also, a similar subset of memory T cells producing IL-17A has been shown to accumulate in kidneys in some hypertensive mouse models (27–29), including the development of l-NAME/high-salt hypertension and glomerular injury (27).

The endothelial surface layer is the first contact point between circulating immune cells and the local tissue environment, via its glycocalyx that is composed of a glycoprotein-polysaccharide complex including heparan sulfate and HA (30). HA is the major ligand of the CD44 receptor (17). Activated T cells are phenotypically defined by their expression of high levels of CD44 (31). We present evidence supporting the notion that activated memory T cells preferentially home into glomerular capillaries via direct binding of CD44 on the surface of the T cells to HA within the endothelial glycocalyx (Figure 4, Figure 5, and Figure 6). The glomerular intravascular homing of these activated memory T cells is substantially enhanced by the glomerulus-specific increase in endothelial surface glycocalyx and its HA content, particularly in LN (Figure 5 and Figure 6). This interplay between homing activated memory T cells and the accumulated glomerular endothelial glycocalyx via its HA component represents a potentially novel mechanism driving LN, distinctive from at least some other inflammatory kidney diseases, although similar to previously described CD44-HA interactions in neutrophil recruitment in other organs, including the liver (32, 33). Whether this same mechanism is at play in other immune and autoimmune forms of GN needs to be established in future studies. The molecular identity of factors contributing to glomerulus-specific glycocalyx and HA accumulation in LN is unclear and needs future investigation, although the l-NAME experiments (Figure 4, A–F) suggested the essential role of endothelial injury. There is a possibility that autoantibodies may contribute to HA accumulation in the glomerular endothelial glycocalyx during the progression of LN and that the unique phenotype and gene expression profile of the glomerular endothelium (34) may make this vascular bed especially responsive to autoantibodies or other circulating factors in LN. Thus, few reports suggest that autoantibodies (especially anti-dsDNA antibodies) could increase de novo synthesis of HA, possibly through increased HA synthase (HAS 2) transcription (35, 36).

It should be noted that because of the iv labeling approach, only the glomerular intravascular homing of CD44^+^ leukocytes was visualized and analyzed in the present study. Intravital MPM imaging found that almost all CD44^+^ cells that homed in glomerular and peritubular capillaries were also CD3^+^ (Supplemental Figure 4), confirming the cytometry-based findings (Figure 2) that activated memory T cells are the major immune cell type in both glomerular and tubulointerstitial kidney compartments of these LN mice. Although many types of leukocytes, including T cells, are known to transmigrate through the endothelium into the interstitium (glomerular mesangium) and play important roles in various immunopathologies (37), these cells were not labeled in our study. Therefore, our calculations likely underestimated the actual immune cell homing.

According to the current paradigm, the glomerular endothelial glycocalyx is an important protective layer of the glomerular filtration barrier. Kidney injury models such as 5/6 nephrectomy (38), adriamycin nephropathy (39), the MWF spontaneous albuminuria rat model (18), STZ diabetic nephropathy (40), aldosterone treatment (19), and the Zucker fatty rat obese model (41) are characterized by glomerular endothelial glycocalyx shedding causing albumin leakage. Accordingly, MPM imaging has demonstrated that the loss of endothelial glycocalyx causes albumin leakage in MWF rats (18) and aldosterone treatment models (19). In contrast, we show here, for the first time to our knowledge, that in LN there is a robust accumulation of glomerular endothelial glycocalyx and its specific molecular component HA (Figure 5 and Figure 6). Normal healthy C57BL/6 WT, C57BL/6.BAFFTg, and NZM WT mice (4–6 weeks) exhibited 0.5~0.6 μm thickness of endothelial glycocalyx, similar to previous studies in healthy animals (18) and comparable with results obtained with electron microscopy (42–44). In agreement with previous studies we also show that in STZ and NOD models of diabetic nephropathy, there is a decrease in glomerular endothelial glycocalyx (Figure 6 and Supplemental Figure 5) (45). Distinctively, both LN mouse lines, the NZM WT (7–9 months) and the NZM.BAFFTg mice (4–6 weeks), showed ~3.5-fold increase in endothelial glycocalyx thickness (Figure 5). Interestingly, while LN-specific glomerular endothelial glycocalyx accumulation was associated with increased glomerular albumin leakage (Figure 3) and proteinuria (Figure 7P), and glycocalyx removal by H treatment improved albumin leakage (Figure 7L) and proteinuria (Figure 7P), the opposite change in glomerular endothelial glycocalyx (i.e., reduction) was associated with glomerular albumin leakage in NOD mice, another model of proteinuria (Supplemental Figure 5, E and F). These findings suggest that alterations in glomerular endothelial glycocalyx per se are not always directly related to proteinuria development and underscore the importance of additional pathogenic factors (such as immune cell–mediated local inflammation as further discussed below). Furthermore, we demonstrate that the principal glycocalyx component HA was significantly increased in glomeruli of NZM.BAFFTg as evaluated with HABP (Figure 6). These data suggest that glycocalyx accumulation in LN includes an increase in its HA component. Measurements of plasma HA levels found increased levels in LN mice that returned to baseline after H treatment (Supplemental Figure 5H), consistent with the known shedding of HA and other glycocalyx components into the plasma in patients with LN (35, 46). However, systemic shedding of glycocalyx components, including HA, in these LN mice likely did not reach levels high enough to saturate immune cells’ CD44 receptors and to prevent their glomerular homing or to cause clogging of glomerular capillaries. The even and linear rather than patchy profile of FITC-WGA signal (Figure 5 and Supplemental Figure 5), the preserved plasma and red blood cell flow through the glomerular capillaries, and the ability of CD3^+^CD44^+^ T cells to home into glomerular capillaries and the downstream peritubular capillaries (Supplemental Figure 4 and Supplemental Video 1) further support the lack of glomerular clogging. Acknowledging the well-documented difficulties of measuring endothelial glycocalyx in humans (47), translating our findings in spontaneous LN mice to patients with LN will have to be postponed until development of better technical methods suitable for human kidney biopsies.

MPM imaging of glomeruli in vivo in the intact mouse kidney has been used to study glomerular disease mechanisms (6), including dynamics of immune cell surveillance (48). Consistent with the behavior of neutrophils and monocytes in other inflammatory conditions (48), the present study found mostly static or slowly migrating activated memory T cells that homed into glomeruli (Supplemental Videos 1, 2, and 6). The intravital MPM approach and the NZM.BAFFTg mouse model (Figure 3) were essential for surveying and acquiring visual clues on the local glomerular tissue microenvironment in LN in vivo, including the quantitative imaging of glomerular endothelial glycocalyx alterations and immune cell interactions (Figure 4 and Figure 5). The labeling and fate tracking of endogenous immune cells in the same glomerulus over several days add to the in vivo glomerular cell study toolbox that has been established for the MPM imaging technology (6).

Finally, this T cell homing mechanistic process can be specifically manipulated by the enzyme H and likely developed further as a specific therapeutic modality in LN. Although the immune cell arm of the CD44-HA interaction could also be therapeutically manipulated (e.g., by reducing CD44 expression via targeting its regulatory pathways, such as pERM and ROCK, ref. 49), the present study focused on targeting HA on the endothelium. H degrades HA glycosaminoglycans from the glycocalyx (50). Also, it has been established that H injection does not affect glomerular charge selectivity and does not cause significant increases in the glomerular permeability of macromolecules (51). We show here by the reduced FITC-WGA lectin fluorescence intensity that H significantly diminished the accumulated glomerular endothelial glycocalyx in LN and efficiently reduced its HA content as demonstrated by reduced HABP labeling (Figure 5 and Figure 6). Consequently, acute iv H injection reduced the number of activated memory T cells homed into glomeruli by approximately 40% within 1 hour (Figure 4R). We demonstrate a dose-dependent effect of H, with an IC_50_ of about 20 U, in reducing the homed activated memory T cells in glomeruli of LN mice, and causing significant improvement in glomerular albumin leakage (Figure 7, A–L). A short-course regimen of iv H injections caused an almost complete elimination of activated memory T cells from glomeruli and a cumulative reduction in albuminuria up to 50 days posttreatment (Figure 7, P and Q), despite the short half-life of H (terminal half-life in plasma is approximately 40 minutes, ref. 52). Endothelial glycocalyx targeting and specificity of H treatment were confirmed by the similar effects of treatment with heparinase III, an alternative glycocalyx-degrading enzyme (53) (Figure 7, P and Q; and Supplemental Figure 5). Remarkably, the short-course enzymatic removal of glomerular endothelial glycocalyx was long-lasting in LN mice without significant glycocalyx recovery (Supplemental Figure 5), despite its established high turnover (30). Considering that glomerular endothelial glycocalyx degradation has previously been linked to pathology development, the improvement in glomerular albumin leakage in response to glycocalyx removal in the present study seems paradoxical. However, the LN-specific condition (glycocalyx accumulation), the efficient removal of homed T cells, and therefore the diminished inflammation are the likely reasons for the protective effects of H treatment. In addition to long-lasting glycocalyx removal, the prolonged effect of H can be further explained, at least in part, by the fact that, in addition to glomeruli, H also reduced accumulation of activated memory T cells in other target organs (e.g., skin, skeletal muscle, spleen, peritoneum) (Supplemental Figure 6A). Although not as strong as in the kidney glomeruli, the presence of endothelial surface glycocalyx in nonrenal vascular beds, such as in the spleen (Supplemental Figure 6B), suggests similar CD44 and HA-mediated mechanisms of T cell homing in these organs. Thus, H might modulate the overall bioavailability of these T cells by affecting their distribution throughout the entire circulation in the whole animal (54). Accordingly, the present experimental approach has high potential impact for future therapeutic development and may be appropriate not only for LN but also for SLE.

## Methods

### Mice.

All mice were produced and bred at the University of Southern California (USC) and were maintained in specific pathogen–free quarters, and all animal protocols were approved by the USC Institutional Animal Care and Use Committee. The NZM.2328 recombinant inbred mouse strain (7) was used to produce a variety of Tg and KO lines on this lupus background. In the present study we used female mice from NZM.2328 WT (NZM WT), NZM.IRAK1^–/–^, IFN receptor–α (NZM.IFNAR^–/–^), NZM.BAFF^–/–^, TNF receptor p55/p75–double deficient (TNFR-DKO), NZM.Stat4^–/–^, and NZM.Stat6^–/–^ mice that were previously described (8–12, 15). The BAFFTg mice driven by liver-specific alpha1-antitrypsin promoter (15) on the C57BL/6 background were backcrossed onto the NZM.2328. Introgression of the genotypes was accelerated by a marker-assisted selection protocol using microsatellite markers spanning the entire genome and including markers to ensure that all known SLE susceptibility loci remained intact (55). Diabetes was induced by streptozotocin injection (50 μg/g BW, i.p.) once a day for 5 consecutive days. NOD mice were provided by Sarah Hamm-Alvarez at USC.

### Quantitative real-time PCR.

Total RNA was extracted with RNeasy Mini Kit (QIAGEN), and cDNA was prepared with the iScript cDNA synthesis kit (Bio-Rad). Real-time PCR was performed with TaqMan probes and the 7500 Fast Real-Time PCR system (Applied Biosystems, Thermo Fisher Scientific). All samples were normalized to the housekeeping gene GAPDH internal control.

### Intracellular IL-17A and RORgc expression.

Naive and activated memory CD4^+^ spleen cells were isolated from 7- to 9-month-old female NZM WT mice or 7- to 9-month-old C57BL/6 mice by depleting B cells with magnetic beads (Invitrogen Dynal), followed by flow cytometry sorting gated on CD4^+^CD62L^hi^CD44^lo^ (for naive T cells) or CD4^+^CD44^hi^CD62L^lo/neg^ (for activated memory T cells) using a FACSVerse (BD Biosciences). Sorted cells were stimulated with PMA (50 ng/mL) and ionomycin (100 ng/mL) for 5 hours and BFA (5 μg/mL) for 4 hours. In some experiments, these cells were stimulated with anti-CD3/CD28–coated beads (1:5 ratio) for 5 days, and PMA, ionomycin, and BFA were added to cultures for the final 5 hours, as above. Cells were stained for surface CD4, CD62L, and CD44; fixed; permeabilized; and then stained for IL-17A (eBioscience, Thermo Fisher Scientific). Antibody numbers are given in parentheses: IL-17A (TC11-18H10.1, BioLegend), CD4 (L3T4), CD62L (MEL-14), and CD44 (IM7).

To determine the effect of H, activated memory T cells were isolated from 7- to 9-month-old female NZM.2328 WT mice by depleting B cells with magnetic beads followed by flow cytometry sorting gated on CD4^+^CD44^hi^CD62L^lo/neg^. Cells were incubated with or without 50 U (MilliporeSigma, H3506) of H for 1 hour. The experiment was performed twice, each in triplicates. Total RNA was extracted, followed by cDNA preparation, and real-time PCR was performed with TaqMan probes for IL-17A and RORgc, respectively. All data were normalized to the housekeeping gene GAPDH internal control.

### Soluble IL-17A production.

Cells were isolated and stimulated as above but without BFA. Supernatants were harvested, and the soluble IL-17A levels were determined by ELISA (eBioscience, Thermo Fisher Scientific), according to the manufacturer’s instructions.

### Assessment of kidney pathology.

The development of proteinuria was measured using Albustix assay strips (Bayer) with a scale ranging from 0 to 4+. Severe proteinuria was defined as ≥3+ (>300 mg/dL) on 2 consecutive examinations. For the assessment of renal pathology, half of each kidney was fixed in 4% formaldehyde. Paraffin sections were stained with H&E, periodic acid-Schiff, and Masson’s trichrome and scored in a blind fashion (using a 0 to 3 scale) for the following features: glomerular hypercellularity, necrotizing lesions, karyorrhexis, cellular crescents, and hyaline deposits (these features indicate glomerular activity score); interstitial inflammation, tubular cell necrosis, and epithelial cells or macrophages in tubular lumens (tubulointerstitial activity score); and glomerulosclerosis, glomerular scars, fibrous crescents, tubular atrophy, and interstitial fibrosis (chronic lesion score). The scores for individual features were summed to obtain the 3 main scores (glomerular activity score, tubulointerstitial activity score, and chronic lesion score), and then the 3 main scores were summed to obtain a composite KHS as described previously (8, 10).

### Kidney glomerular and tubulointerstitial isolation and flow cytometry.

We used the methodology described and validated by Takemoto et al. (14) with slight modifications. Magnetic Dynabeads M-450 (Invitrogen Dynal) were washed and blocked. Anesthetized mice were perfused with a perfusion system (Automate Scientific) through the heart with 10 mL phosphate-buffered saline (PBS) followed by Dynabeads (4 × 10^7^ beads) diluted in 20 mL PBS that get trapped in the glomeruli. The kidneys were removed, minced into 1–3 mm^3^ pieces, and digested in collagenase (1 mg/mL collagenase D, MilliporeSigma) and 100 U/mL DNase I (Roche Diagnostics) at 37°C for 30 minutes with gentle agitation. The collagenase-digested tissue was gently pressed through a 100 μm strainer twice and then washed with 5 mL PBS. The cell suspension was then centrifuged at 200*g* for 5 minutes. The glomeruli containing Dynabeads were collected by a magnetic particle collector. Both compartments were checked under the microscope to verify the purity of each portion. The glomerular compartment was subject to sequential cycles of dissociation steps using gentleMACS (Miltenyi Biotec) and enzymatic digestion with collagenase P 1 mg/mL (Roche Diagnostics) and 100 U/mL DNase I at 37°C for 20 minutes followed by filtration through a 70 μm strainer. The tubulointerstitial compartment was subjected to 1 cycle of mechanical dissociation using the gentleMACS program C followed by 30 minutes on ice and filtration through a 70 μm strainer to discard clumps of tubules. The separated single-cell suspensions were stained with a mixture of fluorochrome-labeled mAbs specific for murine CD45.2 (clone 104), Mac-2 (M3/38, BioLegend), Ly-6G (1A8, BioLegend), CD3 (145-2C11), CD4 (L3T4), CD8 (53-6.7, eBioscience, Thermo Fisher Scientific), CD25 (PC61), CD19 (6D5), CD44 (IM7), CD62L (MEL-14), CD69 (H1.2F3, BioLegend), and NK1.1 (PK136). Unless otherwise mentioned, the antibodies mentioned in Methods were purchased from BD Biosciences. Matched fluorochrome-labeled IgG isotypes were used as controls. In some experiments in parallel to the kidney compartment, flow cytometry of splenocytes was performed from the same animal so that lymphocyte subsets in the kidney could be compared with similar subsets in the spleen of the same animal. Flow cytometry was performed on FACSCalibur or FACSVerse and analyzed using FlowJo (Tree Star).

### Competitive inhibition of CD44 and HA binding.

To test the specificity of binding via HA, activated memory T cells in RPMI 1640 and 10% FBS were exposed to a saturating dose of 1 mg/mL of HA (MilliporeSigma) for 30 minutes. Hybridomas producing HA-blocking rat anti–mouse CD44 (KM81) (56) were provided by Katalin Mikecz (Rush University Medical Center, Chicago, Illinois, USA). Activated memory T cells were incubated with KM81 (100 μg, 1 μg, and 10 ng) for 30 minutes, washed, and then injected into NZM.BAFFTg mice iv via retro-orbital sinus.

### Intravital serial MPM.

Under continuous anesthesia (isoflurane 1%–4% inhalant via nose cone), the animals were placed on the stage of the inverted microscope as described previously (57, 58). Body temperature was maintained with a homeothermic blanket system (Harvard Apparatus). Alexa Fluor 594–conjugated bovine serum albumin (Invitrogen, Thermo Fisher Scientific) was injected iv to label the plasma and to detect albumin leakage into Bowman’s space. The images were acquired using a Leica TCS SP5 multiphoton confocal fluorescence imaging system with a ×63 Leica glycerol-immersion objective (numerical aperture 1.3) powered by a Chameleon Ultra-II MP laser at 860 nm (Coherent) and a DMI 6000 inverted microscope’s external non-descanned hybrid detectors (Leica Microsystems) equipped with FITC/TRITC emission filters. The potential toxicity of laser excitation and fluorescence to the cells was minimized by using a low laser power and high scan speeds to keep total laser exposure as minimal as possible. Then, 1.5 μm *Z*-stack images were taken of all superficial glomeruli. The usual image acquisition consisted of only 1 *Z*-stack per glomerulus (<3 minutes), which resulted in no apparent cell injury. Serial imaging of the same glomerulus in the same animal/kidney was performed until day 5 after the first imaging session. The detailed method of serial survival imaging with surgical kidney exteriorization was described before (5). Regarding time-lapse imaging, we selected superficial glomeruli, and time (*xyt*) series with 1 frame per second were recorded before and after H treatment.

### Glomerular sieving coefficient.

Alexa Fluor 594 bovine serum albumin was used to evaluate albumin leakage into Bowman’s space. Regions of interest were drawn in glomerular capillary plasma and Bowman’s space, and image analysis was performed as described previously (18, 20).

### Induction of endothelial damage by l-NAME and high-salt diet.

Female NZM WT mice (4 weeks old) were randomly assigned to one of 2 experimental groups: vehicle (normal water) control, or l-NAME, 1 g/L (MilliporeSigma), in drinking water and 4% high-salt diet (Harlan, now Envigo, TD92012). Additional control female NZM.BAFFTg (3–4 weeks old) mice were given normal water and diet. All treatments were continued for 2 weeks. Blood pressure was measured via tail-cuff and MPM imaging performed as above. Flow cytometry–purified and –sorted splenic activated memory T cells from 7- to 9-month-old NZM WT females (1 × 10^6^ cells/100 μL) were injected iv via retro-orbital sinus. One hour after injection, the number of activated memory T cells in glomeruli were calculated in 3D volume (*xyz*) images.

### In vivo labeling of endogenous CD3^+^CD44^+^ T cells.

Alexa Fluor 594–labeled anti-CD3 antibody (BioLegend) and Alexa Fluor 488–labeled anti-CD44 antibody (BioLegend) were used to detect activated memory T cells and directly and quantitatively visualize their glomerular intravascular homing in C57BL/6, NZM WT, and NZM.BAFFTg mice (3–4 weeks old) using serial MPM. These antibodies were administered via retro-orbital sinus each at a dose of 30 μL. Clearly positively labeled cells could be seen immediately after antibody injection. The numbers of CD3 and CD44 double-positive cells in entire glomeruli were calculated in 3D volume (*xyz*) images before and after H treatment. Multiple glomeruli per each mouse were analyzed as indicated to avoid sampling errors. Positively labeled cells could be identified in skin, muscle, peritoneal membrane, and spleen and enumerated in single *xy* images before and after H treatment.

### Physiologic and biochemical measurements.

Systolic arterial blood pressure was measured by the tail-cuff method (BP-2000 Blood Pressure Analysis System, Visitech Systems). Spot urine was collected from animals, and urine albumin was measured by using murine microalbuminuria ELISA kit (Albuwell M kits, Exocell). Urine creatinine was measured via microplate assay (The Creatinine Companion, Exocell), and ACR was calculated.

### H and heparinase III treatment.

NZM.BAFFTg mice were given a bolus of PBS (0.02 mL iv via retro-orbital sinus) or H (0.2, 2.0, 20.0, 50.0, and 200 U, MilliporeSigma, H3506) in PBS. Glomeruli were evaluated 1 hour after H administration based on previous reports that H effect on capillary glycocalyx was maximal at this time point (59, 60). With serial in vivo MPM imaging, single H treatment (200 U) was used on day 1, day 3, and day 5 (total 3 times injection), and the number of CD3^+^ and CD44^+^ cells of the same glomerulus was measured before and after H treatment. In some experiments the alternative glycocalyx-degrading enzyme heparinase III (MilliporeSigma) was injected iv (0.7 U/mouse) as above.

### Endothelial surface layer imaging.

FITC-WGA lectin (*Triticum vulgaris*; L4895, MilliporeSigma), administered via retro-orbital sinus at 2 μg/g body weight, was used to visualize the entire glomerular endothelial glycocalyx. FITC-WGA lectin–positive region of the glomerular endothelial capillary surface was visible immediately after injection. FITC-WGA lectin fluorescence intensity and thickness were evaluated before and 1 hour after H treatment (20 U). Fluorescence intensity images were analyzed with LAS AF software (Leica) as previously described (18). For calculation of FITC-WGA lectin intensity, the signal was normalized to the green autofluorescence from proximal tubular cells. Quantification of glycocalyx thickness was performed on line profile by calculating the width of FITC-WGA signal at half-maximum fluorescence intensity as shown in Figure 5A and as described before (18, 19, 61, 62).

### HA content of endothelial glycocalyx and plasma.

This assay was performed on 7 μm frozen kidney tissue sections after PBS infusion at sacrifice. Sections were labeled with HABP (recombinant human versican G1 domain expressed in *E. coli*, AMSBio). HABP was conjugated with Alexa Fluor 594 (Invitrogen, Thermo Fisher Scientific) as per the manufacturer’s instructions. Tissue sections were incubated with Alexa Fluor 594 HABP overnight at a dilution of 1:25 and were examined immediately by using a Leica TCS SP5 confocal microscope. The HABP-positive area was quantified as percentage of each glomerulus with ImageJ software (NIH). HABP fluorescence intensity was analyzed by LAS AF software.

Plasma HA content was measured using the DHYAL0 Hyaluronan Quantikine ELISA Kit (R&D Systems, Bio-Techne) according to the manufacturer’s instructions.

### Statistics.

Data are reported as means ± SEM, with *n* referring to the number of animals studied. Paired or unpaired Student’s *t* tests (2 tailed) or 1-way ANOVA with Tukey’s multiple-comparisons test was performed using GraphPad Prism 6. *P* < 0.05 was considered significant. Spearman’s correlation analysis was used to determine the correlation between parameters.

### Study approval.

All animal protocols were approved by the Institutional Animal Care and Use Committee at the USC.

## Author contributions

HK, NY, IMS, ARB, GG, and COJ conducted the experiments; DD, KK, and MJB designed and established new experimental approaches. COJ and JPP designed the study and wrote the paper.

## Supplementary Material

Supplemental data

Supplemental Video 1

Supplemental Video 2

Supplemental Video 3

Supplemental Video 4

Supplemental Video 5

Supplemental Video 6

Supplemental Video 7

Supplemental Video 8

## Version 1 09/01/2020

In-Press Preview

## Version 2 10/02/2020

Electronic publication

## References

[1] Anders HJ, Rovin B (2016). A pathophysiology-based approach to the diagnosis and treatment of lupus nephritis. Kidney Int.

[2] Yu F, Haas M, Glassock R, Zhao MH (2017). Redefining lupus nephritis: clinical implications of pathophysiologic subtypes. Nat Rev Nephrol.

[3] Saxena R, Mahajan T, Mohan C (2011). Lupus nephritis: current update. Arthritis Res Ther.

[4] Tsokos GC, Lo MS, Costa Reis P, Sullivan KE (2016). New insights into the immunopathogenesis of systemic lupus erythematosus. Nat Rev Rheumatol.

[5] Hackl MJ (2013). Tracking the fate of glomerular epithelial cells in vivo using serial multiphoton imaging in new mouse models with fluorescent lineage tags. Nat Med.

[6] Peti-Peterdi J, Kidokoro K, Riquier-Brison A (2015). Novel in vivo techniques to visualize kidney anatomy and function. Kidney Int.

[7] Rudofsky UH, Lawrence DA (1999). New Zealand mixed mice: a genetic systemic lupus erythematosus model for assessing environmental effects. Environ Health Perspect.

[8] Jacob CO (2003). Pivotal role of Stat4 and Stat6 in the pathogenesis of the lupus-like disease in the New Zealand mixed 2328 mice. J Immunol.

[9] Waters ST (2001). NZM2328: a new mouse model of systemic lupus erythematosus with unique genetic susceptibility loci. Clin Immunol.

[10] Jacob N (2009). Accelerated pathological and clinical nephritis in systemic lupus erythematosus-prone New Zealand Mixed 2328 mice doubly deficient in TNF receptor 1 and TNF receptor 2 via a Th17-associated pathway. J Immunol.

[11] Agrawal H (2009). Deficiency of type I IFN receptor in lupus-prone New Zealand mixed 2328 mice decreases dendritic cell numbers and activation and protects from disease. J Immunol.

[12] Jacob CO (2006). Paucity of clinical disease despite serological autoimmunity and kidney pathology in lupus-prone New Zealand mixed 2328 mice deficient in BAFF. J Immunol.

[13] Jacob CO (2015). Differential development of systemic lupus erythematosus in NZM 2328 mice deficient in discrete pairs of BAFF receptors. Arthritis Rheumatol.

[14] Takemoto M (2002). A new method for large scale isolation of kidney glomeruli from mice. Am J Pathol.

[15] Mackay F (1999). Mice transgenic for BAFF develop lymphocytic disorders along with autoimmune manifestations. J Exp Med.

[16] Baylis C, Mitruka B, Deng A (1992). Chronic blockade of nitric oxide synthesis in the rat produces systemic hypertension and glomerular damage. J Clin Invest.

[17] Aruffo A, Stamenkovic I, Melnick M, Underhill CB, Seed B (1990). CD44 is the principal cell surface receptor for hyaluronate. Cell.

[18] Salmon AH (2012). Loss of the endothelial glycocalyx links albuminuria and vascular dysfunction. J Am Soc Nephrol.

[19] Butler MJ (2019). Aldosterone induces albuminuria via matrix metalloproteinase-dependent damage of the endothelial glycocalyx. Kidney Int.

[20] Nakano D (2012). Multiphoton imaging of the glomerular permeability of angiotensinogen. J Am Soc Nephrol.

[21] Peti-Peterdi J, Sipos A (2010). A high-powered view of the filtration barrier. J Am Soc Nephrol.

[22] de la Motte CA, Drazba JA (2011). Viewing hyaluronan: imaging contributes to imagining new roles for this amazing matrix polymer. J Histochem Cytochem.

[23] Sabzevari H, Propp S, Kono DH, Theofilopoulos AN (1997). G1 arrest and high expression of cyclin kinase and apoptosis inhibitors in accumulated activated/memory phenotype CD4+ cells of older lupus mice. Eur J Immunol.

[24] Chu EB, Ernst DN, Hobbs MV, Weigle WO (1994). Maturational changes in CD4+ cell subsets and lymphokine production in BXSB mice. J Immunol.

[25] Crispin JC (2008). Expanded double negative T cells in patients with systemic lupus erythematosus produce IL-17 and infiltrate the kidneys. J Immunol.

[26] Stadhouders R, Lubberts E, Hendriks RW (2018). A cellular and molecular view of T helper 17 cell plasticity in autoimmunity. J Autoimmun.

[27] Itani HA (2016). CD70 exacerbates blood pressure elevation and renal damage in response to repeated hypertensive stimuli. Circ Res.

[28] Madhur MS (2010). Interleukin 17 promotes angiotensin II-induced hypertension and vascular dysfunction. Hypertension.

[29] Harrison DG (2011). Inflammation, immunity, and hypertension. Hypertension.

[30] Reitsma S, Slaaf DW, Vink H, van Zandvoort MA, oude Egbrink MG (2007). The endothelial glycocalyx: composition, functions, and visualization. Pflugers Arch.

[31] Sprent J, Surh CD (2002). T cell memory. Annu Rev Immunol.

[32] McDonald B, Kubes P (2015). Interactions between CD44 and hyaluronan in leukocyte trafficking. Front Immunol.

[33] McDonald B (2008). Interaction of CD44 and hyaluronan is the dominant mechanism for neutrophil sequestration in inflamed liver sinusoids. J Exp Med.

[34] Barry DM (2019). Molecular determinants of nephron vascular specialization in the kidney. Nat Commun.

[35] Yung S, Tsang RC, Leung JK, Chan TM (2006). Increased mesangial cell hyaluronan expression in lupus nephritis is mediated by anti-DNA antibody-induced IL-1beta. Kidney Int.

[36] Feusi E, Sun L, Sibalic A, Beck-Schimmer B, Oertli B, Wuthrich RP (1999). Enhanced hyaluronan synthesis in the MRL-Fas(lpr) kidney: role of cytokines. Nephron.

[37] Muller WA (2016). How monocytes guard the glomerulus. Proc Natl Acad Sci U S A.

[38] Padberg JS (2014). Damage of the endothelial glycocalyx in chronic kidney disease. Atherosclerosis.

[39] Jeansson M, Bjorck K, Tenstad O, Haraldsson B (2009). Adriamycin alters glomerular endothelium to induce proteinuria. J Am Soc Nephrol.

[40] Satoh M, Kobayashi S, Kuwabara A, Tomita N, Sasaki T, Kashihara N (2010). In vivo visualization of glomerular microcirculation and hyperfiltration in streptozotocin-induced diabetic rats. Microcirculation.

[41] Kuwabara A, Satoh M, Tomita N, Sasaki T, Kashihara N (2010). Deterioration of glomerular endothelial surface layer induced by oxidative stress is implicated in altered permeability of macromolecules in Zucker fatty rats. Diabetologia.

[42] Chappell D (2009). The glycocalyx of the human umbilical vein endothelial cell: an impressive structure ex vivo but not in culture. Circ Res.

[43] Hjalmarsson C, Johansson BR, Haraldsson B (2004). Electron microscopic evaluation of the endothelial surface layer of glomerular capillaries. Microvasc Res.

[44] van den Berg BM, Vink H, Spaan JA (2003). The endothelial glycocalyx protects against myocardial edema. Circ Res.

[45] Jeansson M, Granqvist AB, Nystrom JS, Haraldsson B (2006). Functional and molecular alterations of the glomerular barrier in long-term diabetes in mice. Diabetologia.

[46] Kim KJ, Kim JY, Baek IW, Kim WU, Cho CS (2015). Elevated serum levels of syndecan-1 are associated with renal involvement in patients with systemic lupus erythematosus. J Rheumatol.

[47] Michel CC, Curry FR (2009). Glycocalyx volume: a critical review of tracer dilution methods for its measurement. Microcirculation.

[48] Devi S (2013). Multiphoton imaging reveals a new leukocyte recruitment paradigm in the glomerulus. Nat Med.

[49] Li Y (2007). Phosphorylated ERM is responsible for increased T cell polarization, adhesion, and migration in patients with systemic lupus erythematosus. J Immunol.

[50] Stern R, Kogan G, Jedrzejas MJ, Soltes L (2007). The many ways to cleave hyaluronan. Biotechnol Adv.

[51] Sverrisson K, Axelsson J, Rippe A, Asgeirsson D, Rippe B (2014). Dynamic, size-selective effects of protamine sulfate and hyaluronidase on the rat glomerular filtration barrier in vivo. Am J Physiol Renal Physiol.

[52] Muckenschnabel I, Bernhardt G, Spruss T, Buschauer A (1998). Pharmacokinetics and tissue distribution of bovine testicular hyaluronidase and vinblastine in mice: an attempt to optimize the mode of adjuvant hyaluronidase administration in cancer chemotherapy. Cancer Lett.

[53] Yen W (2015). Endothelial surface glycocalyx can regulate flow-induced nitric oxide production in microvessels in vivo. PLoS One.

[54] Kuipers HF (2016). Hyaluronan synthesis is necessary for autoreactive T-cell trafficking, activation, and Th1 polarization. Proc Natl Acad Sci U S A.

[55] Jacob CO (2013). Development of systemic lupus erythematosus in NZM 2328 mice in the absence of any single BAFF receptor. Arthritis Rheum.

[56] Hutas G, Bajnok E, Gal I, Finnegan A, Glant TT, Mikecz K (2008). CD44-specific antibody treatment and CD44 deficiency exert distinct effects on leukocyte recruitment in experimental arthritis. Blood.

[57] Kang JJ, Toma I, Sipos A, McCulloch F, Peti-Peterdi J (2006). Quantitative imaging of basic functions in renal (patho)physiology. Am J Physiol Renal Physiol.

[58] Kaverina NV (2017). Tracking the stochastic fate of cells of the renin lineage after podocyte depletion using multicolor reporters and intravital imaging. PLoS One.

[59] Henry CB, Duling BR (1999). Permeation of the luminal capillary glycocalyx is determined by hyaluronan. Am J Physiol.

[60] VanTeeffelen JW, Brands J, Jansen C, Spaan JA, Vink H (2007). Heparin impairs glycocalyx barrier properties and attenuates shear dependent vasodilation in mice. Hypertension.

[61] Tsvirkun D, Grichine A, Duperray A, Misbah C, Bureau L (2017). Microvasculature on a chip study of the endothelial surface layer and the flow structure of red blood cells. Sci Rep.

[62] Yen WY, Cai B, Zeng M, Tarbell JM, Fu BM (2012). Quantification of the endothelial surface glycocalyx on rat and mouse blood vessels. Microvasc Res.

