# Supplementary material for: Essential role and therapeutic targeting of the glomerular endothelial glycocalyx in lupus nephritis
Source: JCI Insight. 2020 Oct 2;5(19):e131252. doi: 10.1172/jci.insight.131252 (PMC7566710; doi:10.1172/jci.insight.131252)
Supplement: Supplemental data [file jciinsight-5-131252-s018.pdf]

Supplementary Fig. 1

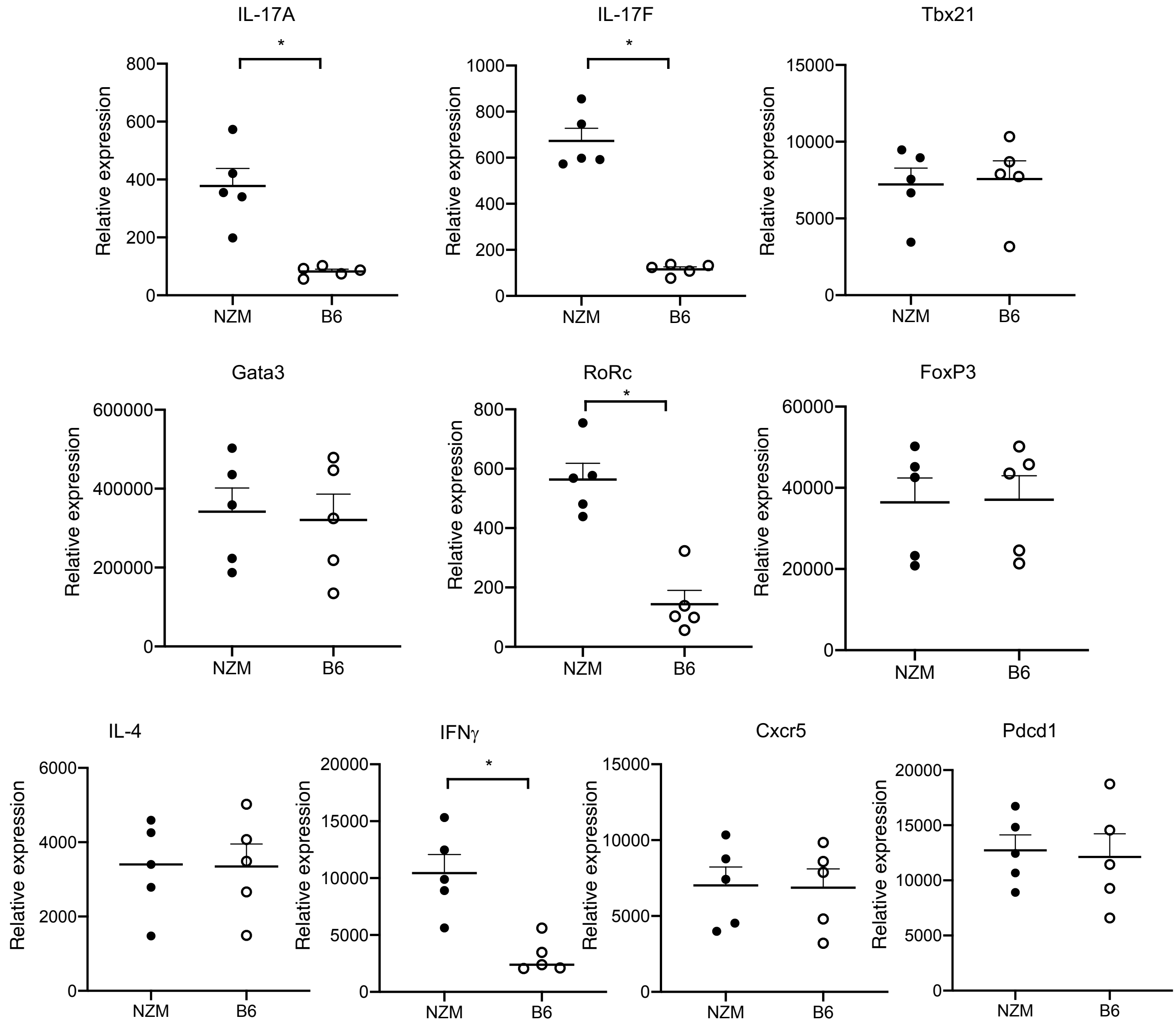

Supplementary Figure 1. The mRNA analysis of activated memory T cells from NZM WT mice reveal a Th17.1 cell phenotype. Real time PCR analysis of activated memory T cells isolated from spleens of 7-9 months-old female NZM WT mice, and age and sex-matched C57BL/6 mice by depleting B cells with magnetic beads (Dynal Biotech), followed by flow cytometry sorting gated on $CD4^{+}CD44^{high}CD62L^{low/neg}$. Graphs show relative expression levels of the indicated cell subset-specific signature genes. Mean ±SEM of 5-7 data points, each of which was derived from material pooled from 2-3 mice. Only statistically significant differences are marked. * p<0.01, based on using unpaired students t-test.

Supplementary Fig. 2

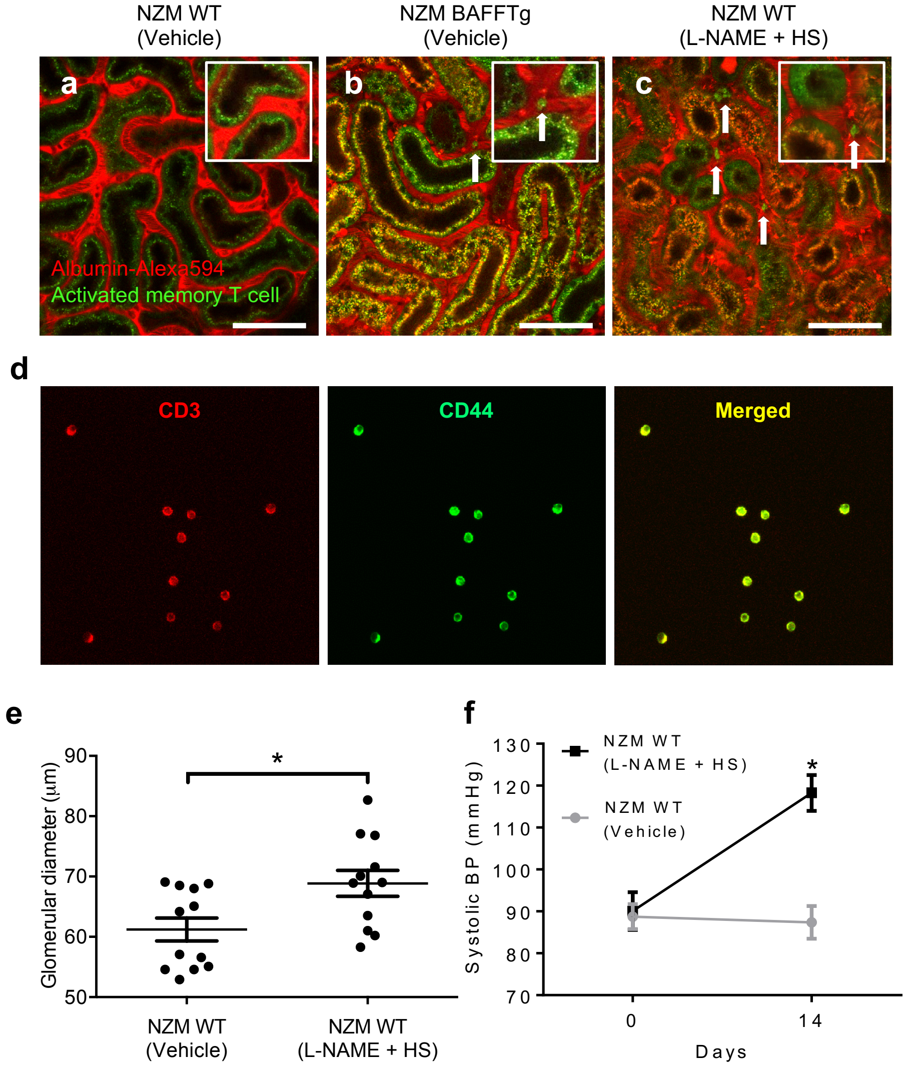

Supplementary Figure 2. MPM imaging of FACS sorted and iv injected splenic activated memory T cells. Intravascular space (plasma) was labeled with iv injected albumin-Alexa594 (red). 4-6 wk old female NZM WT (**a,c**) and NZM.BAFFTg (**b**) mice were treated with L-NAME and high salt diet (HS) (**c**) or vehicle (**a,b**) followed by FACS sorted splenic activated memory T cells iv injection. (**a**-**c**) Representative images of the tubulointerstitium after the injection of activated memory T cells (green, arrows). Bar is 50 µm. (**d**) Fluorescence images and overlay of Alexa594-CD3 (red) and Alexa488-CD44 (green) labeling of T cells. All cells were double+. Comparison of (**e**) glomerular diameter and (**f**) systolic blood pressure in NZM WT Vehicle and L-NAME + high salt treated mice (n=12 glomeruli from n=4 mice each). Data are expressed as means ± SEM, * $p<0.05$, based on using paired and unpaired students t-test.

Supplementary Fig. 3

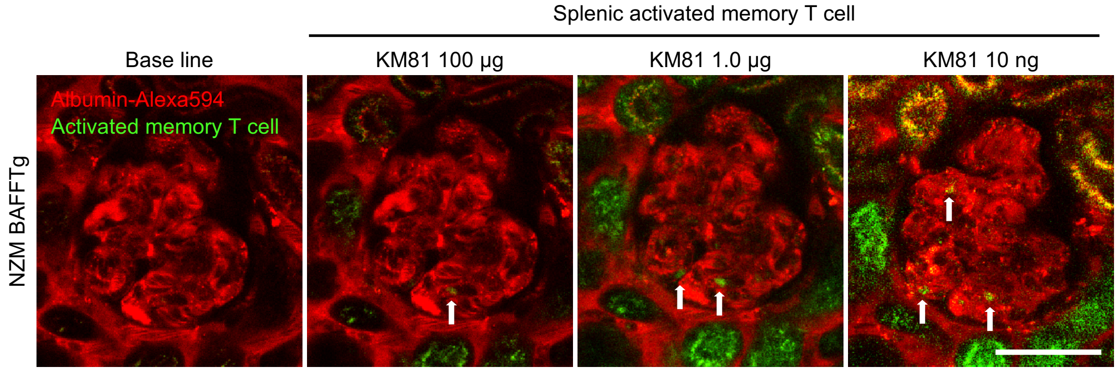

Supplementary Figure 3. MPM imaging of the same single glomerulus from a NZM.BAFFTg mouse before and after iv injection of FACS sorted splenic activated memory T cells that were preincubated with anti-CD44 mAb. Intravascular space (plasma) was labeled with iv injected albumin-Alexa594 (red). Representative images of the same glomerulus after injection of activated memory T cells (green, arrows) that were preincubated with different doses of KM81 anti-CD44 mAb. Increasing doses of KM81 blocked T cell glomerular homing indicated by their reduced number in glomerular capillaries. Bar is 50 µm.

Supplementary Fig. 4

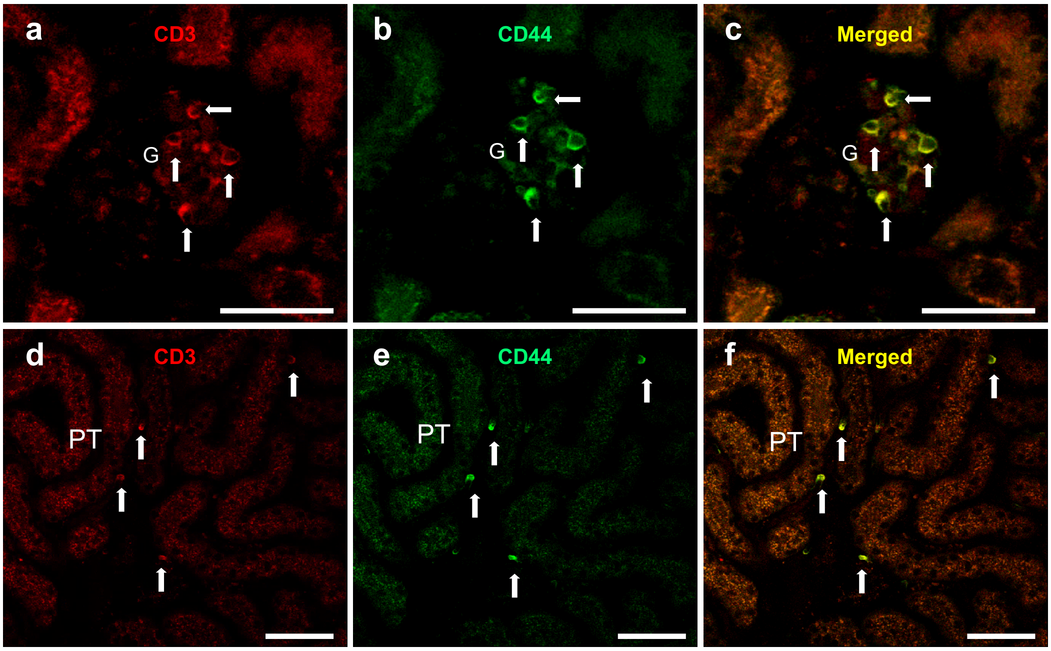

Supplementary Figure 4. Fluorescence labeling and *in vivo* MPM imaging of endogenous CD44⁺, CD3⁺ T cells in NZM.BAFFTg mice. Intravascular space (plasma) was not labeled. (**a**-**f**) Representative images showing endogenous T cells labeled after iv injected Alexa488-conjugated anti-CD44 (green arrows) and Alexa594-conjugated anti-CD3 (red arrows) antibody in glomeruli (**a**-**c**) and tubulointerstitial space (**d**-**f**). Note the co-labeling of most cells for both $CD3^+$ and $CD44^+$ (**c**,**f**). Bar is 50 μm. G, glomerulus. PT, proximal tubule.

Supplementary Fig. 5

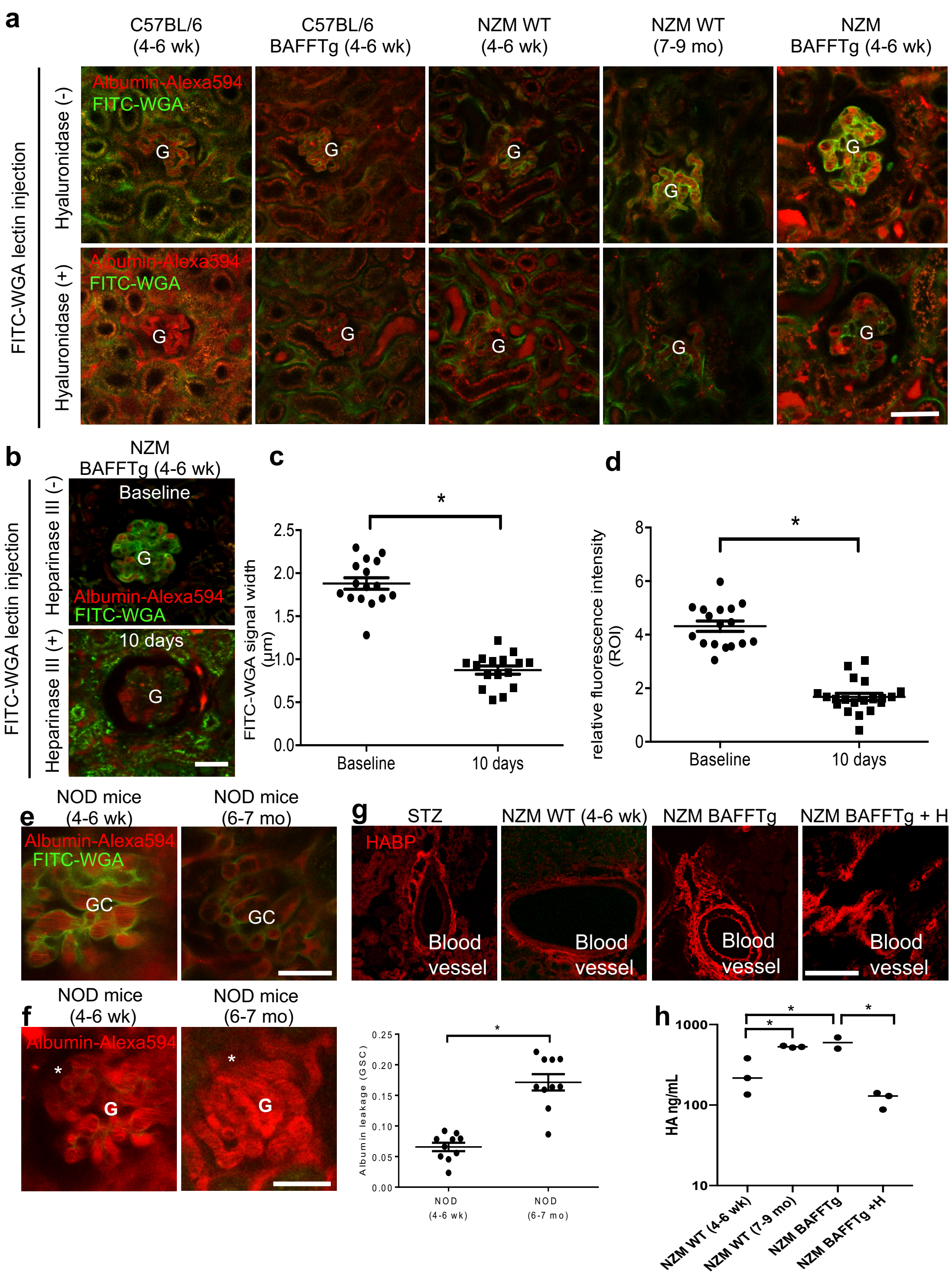

Supplementary Figure 5. Fluorescence imaging of the glomerular endothelial glycocalyx and renal vascular HA content. Intravascular space (plasma) was labeled with iv injected albumin-Alexa594 (red). (**a**) Representative intravital MPM images of the same glomeruli after iv injection of FITC-WGA lectin (green), and before (upper panel) and 1 hour after (lower panel) iv treatment with H enzyme. (**b**) Representative images of endothelial glycocalyx labeling using FITC-WGA lectin (green) before and 10 days after heparinase III treatment (0.7 Units iv). (**c-d***)* Quantification of FITC-WGA signal width (index of endothelial glycocalyx thickness) (**c**) and FITC-WGA lectin fluorescence intensity (**d**) before and 10 days post-heparinase III treatment (n=16 glomeruli from n=4 mice each). Labeling of the tubular epithelium (green) at day 10 confirms the presence of detectable FITC-WGA lectin, but low levels of glomerular glycocalyx. (**e**) Representative images of glomerular endothelial glycocalyx after FITC-WGA injection in young (4-6 wk) and diabetic (6-7 mo) NOD mice. (**f**) Evaluation of glomerular albumin leakage (glomerular sieving coefficient, GSC) into the Bowman's space (asterisk) by MPM imaging in young (4-6 wk) and diabetic (6-7 mo) NOD mice (n=4 mice each group). G, glomeruli. (**g**) Representative images of the specific HA content of blood vessels in different mouse models on histological sections labeled by Alexa594-conjugated HA binding peptide (HABP, red). (h) Plasma HA levels in untreated and H treated (short-course H treatment with a total of 3 iv injections given every other day, 200 U each) lupus mouse strains measured by ELISA (n=3 each). Data are expressed as means ± SEM, * $p<0.05$, based on using unpaired students t-test (c-f) or one-way ANOVA followed by Tukey's multiple comparison test (h). G: glomeruli, GC: glomerular capillary, NOD: non-obese diabetic. STZ: streptozotocin-induced diabetic mice. H, hyaluronidase. Bars are 50 µm.

Supplementary Fig. 6

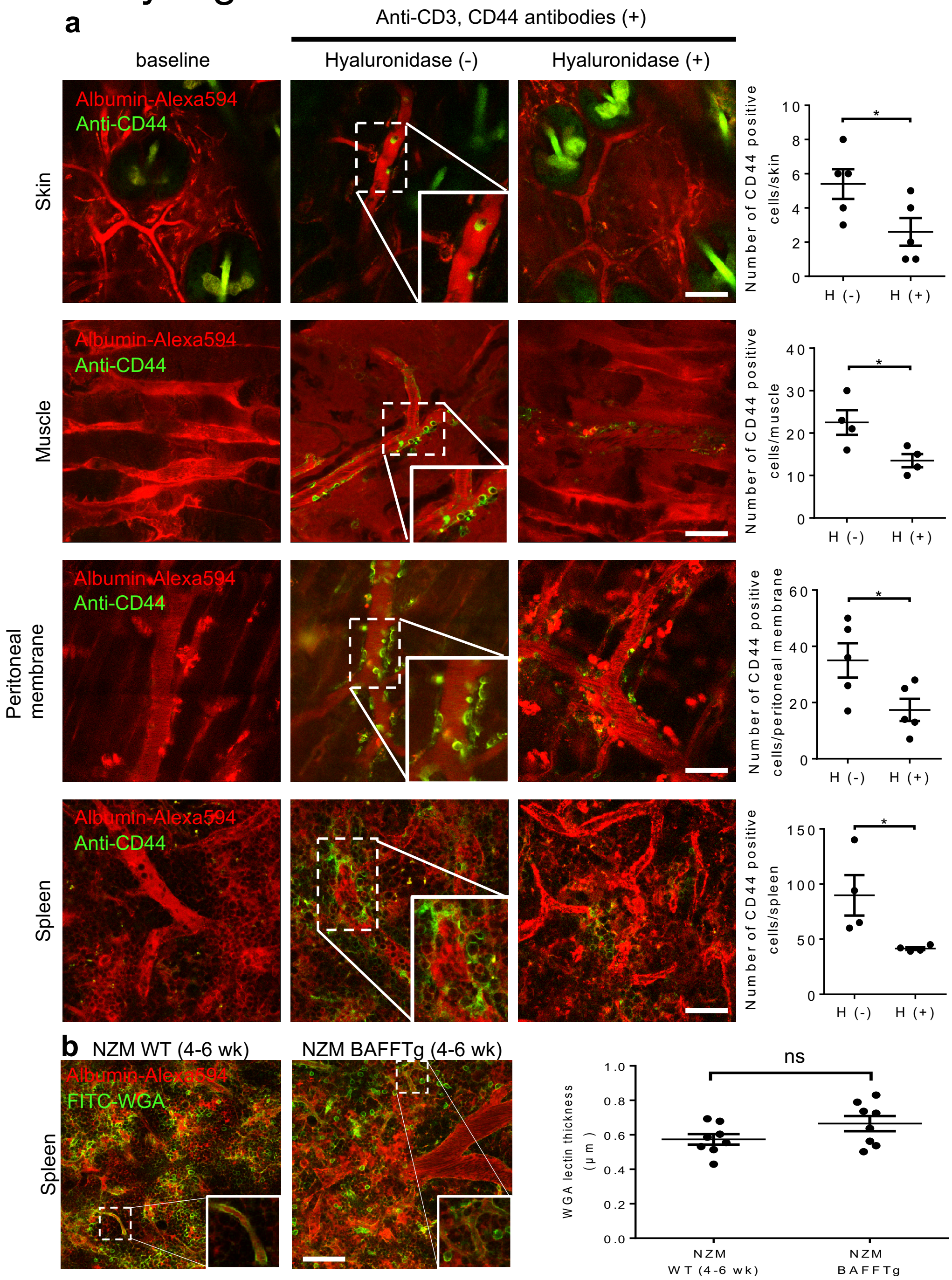

Supplementary Figure 6. Fluorescence images of endogeneous CD3⁺CD44⁺ T cells in skin, skeletal muscle, peritoneum, and spleen of NZM.BAFFTg mice. Alexa488-conjugated anti-CD44 (green) and Alexa594-conjugated anti-CD3 (red) mAb were iv injected with or without H treatment (20 Units iv) as indicated. Intravascular space (plasma) was labeled with iv injected albumin-Alexa594 (red). Cells were double+ for $CD3^+$ and $CD44^+$ (green). Panels on the right summarize the number of $CD44^+$, $CD3^+$ T cells per field in each organ (n=5 areas from n=3 mice each). Skin images also show highly green autofluorescent hair follicles. Center images show magnified insets of $CD44^+$ cell areas as indicated. **(b)** Quantitative MPM imaging of the thickness of vascular endothelial glycocalyx in spleen of NZM WT and NZM BAFFTg mice using iv injected FITC-WGA lectin (green) (n=3 in each group). Data are expressed as means ± SEM, * $p<0.05$, ns: not significant, based on using unpaired students t-test (a-b). H, hyaluronidase. Bars are 50 μm.

SUPPLEMENTARY VIDEO LEGENDS

Supplementary video 1. Optical z-sectioning of a glomerulus with *in vivo* MPM in NZM.BAFFTg mice after anti-CD3 and CD44 mAb injection. Alexa594-albumin (red) was injected iv to label the intravascular space (plasma). Circulating red blood cells appear as dark objects within capillaries. There is no blood flow in focal segmental area of glomerulus. CD3 and CD44 double positive cells are homed not only in the glomerulus but also in peritubular capillaries.

Supplementary video 2. Time-lapse *in vivo* MPM imaging of a glomerulus after anti-CD3 and CD44 mAb injection. Non-specific green autofluorescence is visible in proximal tubular cells. Albumin uptake is visible in circulating inflammatory cells in glomerular capillaries. CD3 and CD44 double positive cells were rolling, homing and migrating in the glomerulus during the 30 minutes of video. The same glomerulus is shown in Figure 4n.

Supplementary video 3. Time-lapse *in vivo* MPM imaging of the same glomerulus as in Supplementary Video 2 after hyaluronidase treatment. The number of CD3 and CD44 double positive cells was significantly reduced after hyaluronidase treatment during the 30 minutes of video. The same glomerulus is shown in Figure 4o.

Supplementary video 4. Serial *in vivo* MPM imaging in a NZM.BAFFTg mouse after FITC-WGA lectin injection. Top-to-bottom glomerular optical sections (1.5 µm Z-stack images) are shown. Plasma is labeled red with Alexa594-albumin. Glomerular glycocalyx labeling (green) is robust in NZM.BAFFTg mice. The same glomerulus is shown in Supplementary Figure 5a.

Supplementary video 5. Serial *in vivo* MPM imaging of the same glomerulus as in Supplementary Video 4 after hyaluronidase injection (20 U). The same optical sections (1.5 µm Z-stack images) are shown. Efficient removal of glomerular endothelial glycocalyx by H treatment was confirmed by diminished WGA labeling (green) in the same glomerulus. The same glomerulus is shown in Supplementary Figure 5a.

Supplementary video 6. Serial *in vivo* MPM imaging of glomeruli in NZM.BAFFTg mice before H treatment at baseline. Top-to-bottom glomerular optical sections (1.5 µm Z-stack images) are shown. Plasma is labeled red with Alexa594-albumin. There are numerous CD3 and CD44 double positive cells homed in the glomerulus. The same glomerulus is shown in Figure 7m.

Supplementary video 7. Serial *in vivo* MPM imaging of the same glomerulus as in Supplementary Video 6 after H treatment at day 3. The same optical sections (1.5 μm Z-stack images) are shown. The number of CD3 and CD44 double positive cells was significantly reduced compared to baseline. The same glomerulus is shown in Figure 7n.

Supplementary video 8. Serial *in vivo* MPM imaging of the same glomerulus as in Supplementary Video 6-7 after H treatment at day 5. The same optical sections (1.5 μm Z-stack images) are shown. CD3 and CD44 double positive cells almost completely disappeared. The same glomerulus is shown in Figure 7o.
